# Optimized purification methods for metallic contaminant removal from directly recycled Li-ion battery cathodes

**DOI:** 10.3389/fchem.2023.1094198

**Published:** 2023-02-08

**Authors:** Kae Fink, Paul Gasper, Joshua Major, Ryan Brow, Maxwell C. Schulze, Andrew M. Colclasure, Matthew A. Keyser

**Affiliations:** National Renewable Energy Laboratory, Alliance for Sustainable Energy, LLC, Golden, CO, United States

**Keywords:** Li-ion battery recycling, direct recycling, metallic contamination, black mass, purification

## Abstract

Metallic contaminants pose a significant challenge to the viability of directly recycling Li-ion batteries. To date, few strategies exist to selectively remove metallic impurities from mixtures of shredded end-of-life material (black mass; BM) without concurrently damaging the structure and electrochemical performance of the target active material. We herein present tailored methods to selectively ionize two major contaminants—Al and Cu—while retaining a representative cathode (LiNi_0.33_Mn_0.33_Co_0.33_O_2_; NMC-111) intact. This BM purification process is conducted at moderate temperatures in a KOH-based solution matrix. We rationally evaluate approaches to increase both the kinetic corrosion rate and the thermodynamic solubility of Al^0^ and Cu^0^, and evaluate the impact of these treatment conditions on the structure, chemistry, and electrochemical performance of NMC. Specifically, we explore the impacts of chloride-based salts, a strong chelating agent, elevated temperature, and sonication on the rate and extent of contaminant corrosion, while concurrently evaluating the effects on NMC. The reported BM purification process is then demonstrated on samples of “simulated BM” containing a practically relevant 1 wt% concentration of Al or Cu. Increasing the kinetic energy of the purifying solution matrix through elevated temperature and sonication accelerates the corrosion of metallic Al and Cu, such that ∼100% corrosion of 75 μm Al and Cu particles is achieved within 2.5 hr. Further, we determine that effective mass transport of ionized species critically impacts the efficacy of Cu corrosion, and that saturated Cl^–^ hinders rather than accelerates Cu corrosion by increasing solution viscosity and introducing competitive pathways for Cu surface passivation. The purification conditions do not induce bulk structural damage to NMC, and electrochemical capacity is maintained in half-cell format. Testing in full cells suggests that a limited quantity of residual surface species are present after treatment, which initially disrupt electrochemical behavior at the graphite anode but are subsequently consumed. Process demonstration on simulated BM suggests that contaminated samples—which prior to treatment show catastrophic electrochemical performance—can be recovered to pristine electrochemical capacity. The reported BM purification method offers a compelling and commercially viable solution to address contamination, particularly in the “fine” fraction of BM where contaminant sizes are on the same order of magnitude as NMC and where traditional separation approaches are unfeasible. Thus, this optimized BM purification technique offers a pathway towards viable direct recycling of BM feedstocks that would otherwise be unusable.

## 1 Introduction

As increasing numbers of lithium-ion batteries (LIBs) reach the end of their functional lifetime, recycling has emerged as a critical strategy to both mitigate hazards associated with disposal and recover valuable constituent materials (Chen et al., 2019). In particular, direct recycling of LIBs—which aims to retain the engineered value of the active species by recovering and refurbishing these components in maximally intact form—is gaining traction as an approach to reduce energy and material inputs while maximizing reclamation potential (Gaines et al., 2021).

At present, direct recycling techniques largely utilize shredding as an initial mechanical processing step to liberate active material. While recent work by Thompson et al. suggests that non-destructive disassembly may offer benefits from both a technoeconomic (Thompson et al., 2021) and material recovery (Thompson et al., 2020) perspective, an efficient disassembly strategy for processing batteries of numerous chemistries and states of degradation has yet to be developed at scale (Neumann et al., 2022). Thus, it is anticipated that shredding will remain a central feature of direct recycling for the near future—and will likely remain the pre-processing technique of choice for batteries recovered following mechanical or thermal abuse (e.g., vehicle crashes). Such upstream shredding introduces significant complexity into subsequent direct recycling methods due to contamination in metallic (i.e., non-ionic) form. Contaminants may be derived from the electrode current collectors (Al, Cu); outer pack casing materials (Si, Mg); or the machinery used in processing (Fe).

Al and Cu contaminants are of particular concern, since these are nearly universally present as current collectors and have been reported in shredded battery material (black mass; BM) in high concentrations due to incomplete active material removal. Fink et al. and others have reported largely adverse impacts of metallic Al (Al^0^) and metallic Cu (Cu^0^) impurities on the performance, cyclability, and safety of cathode materials (Mohanty et al., 2016; Zhang et al., 2020; Fink et al., 2022). Specifically, Fink et al. identified that Al^0^ is reactive under cathodic conditions, inducing atypical first-cycle passivation behavior (Fink et al., 2022); similarly, the introduction of metallic Al to cathodes of the form LiNi_x_Mn_y_Co_z_ (NMC) has been shown to reduce Coulombic efficiency and cell capacity, ostensibly through the formation of a resistive surface-oxidized passivation layer (Mohanty et al., 2016). Further, our team has previously described the deleterious impacts of metallic copper on full-cell performance, including excessive Li consumption, rapid capacity fade, and erratic cycling behavior (Fink et al., 2022). It has also been reported that metallic Cu may induce catastrophic cell failure *via* crosstalk-induced internal short, whereby Cu^0^ ionized to Cu^+^ and/or Cu^2+^ may migrate to the anode and initiate Li dendrite growth (Zhang et al., 2020). Finally, the presence of either impurity (Al^0^ or Cu^0^) reduces the overall quality of the recycled material, which is critical to the viability of direct recycling techniques. Contamination in the “fine” fraction of the BM—where contaminant sizes are on the same order of magnitude as the target active material—is particularly problematic, because traditional separation approaches (optical sorting, magnetic separation, froth floatation, etc.) are unviable. Without appropriate purification, this fine fraction cannot be recycled due to the detrimental performance impacts of contamination.

Strategies to address BM contamination have been developed for hydrometallurgical processes, in which all metals including target cathode active materials such as Li, Ni, Co., and Mn are converted to ionic form *via* chemical leaching. Acidic conditions are generally employed to drive the ionization of metallic components in hydrometallurgy due to the high efficiency of leaching (in multiple reports, >99% leaching efficiency for target cathode metals) and high-yield/high-purity subsequent recovery (Chen et al., 2019; Or et al., 2020). From the bulk leachate solution, undesirable impurity cations are typically removed through pH adjustment, such that the impurities precipitate as hydroxide ions (Kang et al., 2010; Joo et al., 2016), or through a combination of solvent extraction and precipitation steps (Nayl et al., 2015). The leaching conditions used in hydrometallurgy are designed to facilitate the facile ionization of metallic species, and thus are generally effective to ionize impurity metals; however, such conditions drive the breakdown of the physicochemical structure of the target cathode active material (typically LiCoO_2_ (LCO); LiMn_2_O_4_ (LMO), or Li(Ni_x_Co_y_Mn_1-x-y_) O_2_—hereafter NMC) through Li^+^/H^+^ substitution and concurrent or subsequent dissolution of the constituent transition metals (Xuan et al., 2019). Such behavior is problematic in the context of direct recycling, and thus new methods are warranted to enable *selective* ionization of contaminants while retaining the active material intact.

It has been suggested that alkaline (pH > 10) aqueous conditions are non-destructive to metal oxides such as LCO (Tsang and Hailey, 2018), and may enable effective ionization of Al^0^ and Cu^0^. However, there has been no known investigation into the rational optimization of matrix and process parameters to enable rapid and complete ionization of these metals without inducing damage to the cathode active material. The ionization (i.e., corrosion) behavior of Al and Cu under strongly alkaline conditions has been less-well-documented than under acidic conditions, and in such reports, metallic stability—rather than corrosion—is typically the desired outcome. Nonetheless, the findings of such studies may be applied in inverse to inform conditions that are maximally corrosive to the contaminants of interest.

The corrosion of Al under alkaline conditions is predominantly anodic-limited (i.e., rate-limited by the oxidation of Al^0^ to Al(OH)_4_^-^) (Pyun and Moon, 2000) and occurs *via* consecutive formation and re-dissolution of Al(OH)_3_ surface film (Tabrizi et al., 1991; Pyun and Moon, 2000). Numerous reports have indicated the positive pH dependence of Al corrosion rate (Tabrizi et al., 1991; Aleksandrov et al., 2003; Reynolds et al., 2009); this relation is logarithmic at short time scales (minutes to hours) (Zhang et al., 2009). Aleksandrov et al. have reported a distinct dependence of sample morphology (powder vs. foil) on kinetic behavior, with the dimensionally larger Al foil pieces exhibiting a distinct “induction period”, or delay in reaching a steady-state reaction rate (Aleksandrov et al., 2003). This trend scales with sample surface area, and has thus been tied to the presence of a passivating surface film, presumably Al(OH)_3_ (Aleksandrov et al., 2003). Dembrowski et al. have explored the impact of multicomponent aqueous matrices on the solubility of this passivating surface film, and in particular, have suggested that Al(OH)_3_ exhibits enhanced solubility in highly concentrated alkaline solution, especially in the presence of other solvated anions such as NO_2_^−^ and NO_3_^−^ (Dembowski et al., 2020)—a finding consistent with that of Reynolds and Reynolds (Reynolds et al., 2009). However, it has been noted that other counter-anions, such as Cl^−^, demonstrate a negligible—or even slightly negative—impact on Al^0^ corrosion rate (Tabrizi et al., 1991).

King et al. have conducted perhaps the broadest analysis of Cu corrosion in alkaline solution, and have examined the effects of complex matrix parameters—including temperature, pH, salinity, and oxygen content—on Cu behavior (King, 1995; King, 2002; King et al., 2010). The solubility of thermodynamically favored Cu species (CuO, Cu_2_O, and the intermediate species Cu(OH)_2_) at pH > 10 is found to increase with both increasing pH and increasing temperature (King, 2002). Further, a higher concentration of O_2_ is associated with a higher corrosion rate (i.e., higher i_corr_) by promoting the corrosion pathway producing semiconducting Cu(II) - rather than insulating Cu(I) - products (King et al., 2010). Both the kinetics of Cu corrosion (i_corr_) and the thermodynamic favorability of corrosion (E_corr_) are found to be strongly linked to the rate of mass transport away from the corroding surface, such that the corrosion rate of Cu in an oxidizing environment is determined by the diffusion rate of Cu in solution (King et al., 2010).

The reported impact of Cl^−^ on Cu corrosion has been somewhat conflicted, primarily due to the fact that the mechanism of Cl^−^ reactivity is strongly tied to solution pH. Cl^−^ is generally considered to be detrimental to Cu^0^ stability, and is commonly associated with both pitting (i.e., localized corrosion) and bulk corrosion of Cu. In an analysis employing moderately alkaline pH (pH 9), the presence of Cl^−^ was found to induce Cu pitting (Lytle and Schock, 2008); such behavior has been supported *via* theoretical analysis, which implies that the lowest pitting potential for copper occurs in a highly concentrated (∼5 M) NaCl and highly alkaline (pH 11) solution (Arjmand and Adriaens, 2012). However, the relationship between pitting potential and Cl^−^ concentration is convoluted at varying pH values (Allah et al., 1991), which King has tied to changes in Cu surface film properties (King, 2002). Specifically, Cl^−^ is believed to drive corrosion through the stabilization of Cu(I) species as complex anions, such as CuCl^2-^ and CuCl_3_^2-^ (King et al., 2010). Under acidic conditions, where Cu(I) formation is thermodynamically favorable, these complex Cu-Cl anions are the predominant species. Under alkaline conditions, though, the formation of Cu(II) species—typically CuCl_2_^−^ and Cu_2_O—is thermodynamically favored, with the latter (Cu_2_O) appearing to predominate at pH > 10 (King, 2002). While the presence of Cl^−^ is found to reduce E_corr_, increasing Cl^−^ concentration may also exacerbate transport limitations governing active corrosion (King et al., 2010). Finally, even on a predominantly Cu_2_O-passivated surface, the presence of Cl^−^ may lead to defects *via* substitution of monovalent Cl^−^ for divalent O^2-^, which can facilitate a less-insulating surface chemistry that is more susceptible to corrosion (King et al., 2010).

In the present work, we aim to exploit matrix conditions associated with the rapid and favorable corrosion of Al^0^ and Cu^0^ in order to remove metallic Al and Cu from BM without adversely impacting the target cathode active material (NMC). Specifically, we have explored approaches to increase both the kinetic corrosion rate and the thermodynamic solubility of Al^0^ and Cu^0^ simultaneously in a highly alkaline aqueous environment and analyzed the impact of these treatment conditions on the structure, chemistry, and electrochemical performance of NMC. We have evaluated the influence of chloride-based salts, a chelating agent, elevated temperature, and sonication on the rate and extent of contaminant corrosion, as well as on the physical and electrochemical properties of NMC. We demonstrate 100% removal of Al and Cu contamination under idealized conditions, and demonstrate half-cell electrochemical capacity recovery from samples containing 1 wt% contaminant (a relevant nominal impurity concentration for practical shredded black mass) (Fink et al., 2022). We have verified that this process does not induce bulk structural or electrochemical changes to the NMC, and begin to explore optimization of surface structuro-chemistry for applications in full cells. Residual species from the treatment solution and surface species evolved by the neutral aqueous post-treatment rinse appear to reduce on the graphite surface, warranting further exploration and optimization of post-treatment conditions. The reported BM purification process, which is conducted at moderate temperature and with low-cost reagents, offers a promising and commercially viable approach to removing adverse contamination and improving the purity of directly recycled battery materials.

## 2 Materials and methods

### 2.1 Al and Cu corrosion experiments

A series of samples were prepared to evaluate the impact of both varying matrix chemistry and process conditions on the corrosion of metallic Al and Cu. All samples were prepared in 50 mL polypropylene (PP) vials at room temperature. To each vial was added 40 mL ultra-pure deionized (DI) H_2_O (18.2 MΩ-cm). For Cu corrosion experiments, the impact of several additional reagents was evaluated; these include potassium chloride (KCl; ACS reagent, 99.0%–100.5%, Sigma-Aldrich) and the aminopolycarboxylic chelating agent diethylenetriaminepentaacetic acid (DTPA; ≥99%, Sigma-Aldrich). Finally, potassium hydroxide (KOH; 99.99% trace metals basis, Sigma-Aldrich) was added to each sample to achieve a nominal pH value of 13.00 ± 0.02 at calibrated room temperature. Samples were mechanically agitated to ensure complete mixing prior to testing.

Each corrosion test was conducted in a fresh PP container with a cap machined to enable air exchange. The container was sited in a sonicator modified with an external heat exchange loop, which enabled both precise temperature control and constant fluid flow to ensure uniform temperature around the sample (Supplementary Figure S1A). Samples were tested both with and without sonication; the bath temperature was adjusted to account for heat produced during tests including sonication.

In all cases, 10 mg of metallic contaminant powder—either Al (<75 μm, ≥99.95% trace metals basis, Aldrich) or Cu (75 μm, 99%, Aldrich)—was added to the container after the liquid matrix had equilibrated to the bath temperature for 10 min. Samples were stirred at 350 rpm using an overhead stirrer equipped with a PTFE impeller.

The rate and extent of Al corrosion was evaluated by tracking sample pH in the unbuffered solution matrix, as each extent of reaction consumes Al^0^ and OH^−^ on a net equimolar basis (Zhang et al., 2009). Similar use of Al corrosion reaction stoichiometry has previously been used to quantify the rate of Al corrosion by e.g. who monitored the rate of H_2_ generation associated with Al^0^ consumption (Aleksandrov et al., 2003). Both the raw measured value of potential difference between sample and pH electrode (mV) and the calibrated pH value are reported, as this enables increased resolution in back calculations of corrosion extent. Calibration of the pH meter was conducted at quantified room temperature before each test using a fresh sample of pH 12.46 buffer solution (Oakton).

The rate and extent of Cu corrosion could not be evaluated *via* pH monitoring, as the corrosion reaction in the presence of a deprotonated aminopolycarboxylic chelating agent (e.g., DTPA) does not consume OH^−^; rather, the Cu^2+^ produced *via* oxidation in the presence of OH^−^ is rapidly and preferentially chelated as Cu(DTPA)^3-^ (Eivazihollagh, 2018). It is assumed that the high conditional stability constant for Cu-DTPA binding (K = 16.4; The Dow Chemical Company, 2021 discussed in detail in Section 3.1.2) will strongly favor the Cu-DTPA binding pathway, and that the impact of OH-consuming reactions (e.g., to form Cu_2_O) is minor such that the impact on bulk solution pH can be neglected. Thus, an alternative method based on colorimetric back-titration was developed to precisely quantify Cu corrosion. Each Cu solution was prepared with a known molar excess of DTPA. At a designated time point, the sample container was removed from the temperature-controlled bath and was quenched in room-temperature water. The unreacted DTPA was then titrated with a secondary metal (Ca^2+^ as Ca(NO_3_)_2_, received as the tetrahydrate Ca(NO_3_)_2_ • 4H_2_O (≥99.0%, Sigma-Aldrich) and prepared as a 0.132 M solution in deionized H_2_O) in the presence of a colorimetric indicator (calconcarboxylic acid; Sigma-Aldrich), which undergoes a visible color change at the equivalence point. Ca^2+^ was selected as the secondary metal due to its significantly lower binding affinity to DTPA (K = 10.6) (The Dow Chemical Company, 2021), such that there is virtually no possibility of Cu displacement by Ca in the chelated complexes. A visual representation of the colorimetric back-titration progression is shown in Supplementary Figure S1B. The volume of titrant added at the equivalence point i.e., when all excess DTPA has reacted with the titrated Ca^2+^ was then correlated to the mass of Cu^2+^ bound to DTPA, which is nearly identical to the mass of ionized Cu^2+^ (The Dow Chemical Company, 2021).

### 2.2 Preparation and treatment of simulated black mass

The corrosion experiments described in Section 2.1 were used to identify optimal parameters for the ionization of Al and Cu in alkaline solution, as will be discussed. To evaluate the efficacy of such treatment on practical contaminant removal and identify any adverse impacts on NMC, samples of LiNi_0.33_Mn_0.33_Co_0.33_O_2_ (NMC-111; Toda, NM-3101) spiked with 0.1, 1, and 5 wt% metallic contaminant (Al or Cu) were prepared; these contaminant-spiked samples are hereafter referred to as “simulated BM”.

Samples of simulated BM containing 1 wt% contaminant (Al and Cu) were treated using optimized parameters for the corrosion of Al and Cu, as established *via* experimental evaluation described in Section 2.1. The contaminant concentration selected (1 wt%) is reflective of contamination levels observed in the fine fraction of industrial shredded BM, as has been previously reported by Fink et al. (2022), and thus is a relevant metric for the viability of the present methods for practical application. For tests with simulated BM, 1 g simulated BM (99 wt% NMC, 1 wt% Al or Cu) was prepared in a matrix of 40 ml DI H_2_O, with pH adjusted to 13.00 ± 0.02 at calibrated room temperature using KOH. Simulated BM samples spiked with Cu additionally contained 2x molar DTPA, relative to the Cu contaminant. Samples were treated at 60°C with sonication and overhead stirring (350 rpm) for 2.5 h. Samples were centrifuged for 10 min (1,440 rpm, 22°C); the supernatant was decanted, and the remaining solid was rinsed with 30 ml of deionized water and re-centrifuged under the same conditions. After again decanting the supernatant, the resulting treated powder was dried overnight (∼8 h) at 105°C.

### 2.3 Physico-electro-chemical evaluation of simulated black mass

X-ray diffraction (XRD) was conducted on pristine NMC-111, simulated BM, and treated-simulated BM powders using a Rigaku Ultima IV diffractometer with CuKα radiation (40 kV, 40 mA) with a scintillation counter detector and 5 mm divergence slit. High-resolution scans were conducted from 10° to 90° 2θ (0.04° step size; 10 s dwell time). Reitveld refinement was conducted using Profex software (Doebelin and Kleeberg, 2015). Refinement was conducted using layered (trigonal α-NaFeO_2_-type R3̅m) (Shinova et al., 2008), spinel cubic Co_3_O_4_-type Fd3̅m and cubic LiMn_2_O_4_-type Fd3̅m (Mosbah et al., 1983), and rock salt (cubic NiO-type Fm3m) phases; all phases indicated without a reference were sourced from the Profex structure database (Doebelin and Kleeberg, 2015).

Inductively coupled plasma mass spectrometry (ICP-MS) was conducted by Huffman Hazen Laboratories (Golden, CO). Sample aliquots were digested in 1:3 HNO_3_:HCl at 120°C until all HCl was fumed off. Samples were diluted with DI H_2_O prior to ICP-MS analysis.

Cathode electrodes were prepared using NMC-111 power (Toda, NM-3101), conductive carbon black (Timcal graphite/Super-P, MTI Corporation), and polyvinylidene difluoride (PVdF) binder (MTI Corporation) in N-methyl pyrrolidone (NMP) solvent (anhydrous, 99.5%, Sigma-Aldrich). Pristine (i.e., impurity-free) cathodes contained 90 wt% NMC-111, 5 wt% carbon black, and 5 wt% PVdF binder. Untreated simulated BM samples contained metallic Al and Cu impurities across a range of practically relevant concentrations (0.1, 1, and 5 wt%), with contaminant weight substituting for the NMC. Treated simulated BM was assumed to be chemically identical to pristine NMC-111, was thus added at 90 wt%. Mixtures with the noted compositions were prepared using a Flacktek planetary mixer, and were cast onto Al foil (15 μm, MTI) using a doctor blade film coater at a loading of ∼11.25 mg/cm^2^ (∼1.81 mA h/cm^2^ nominal capacity). Electrodes were dried under air until complete solvent evaporation was achieved, and were then dried overnight under vacuum at 105°C.

CR2032 coin cells were assembled at room temperature in a glovebox following overnight cathode drying at 105°C. In all cells, 50 μL of Gen 2 electrolyte (1.2 M LiPF6 in ethylene carbonate (EC):ethylmethyl carbonate (EMC) = 3:7 by weight) and Celgard 2325 separator were used. Half cells were prepared with cathode samples (14 mm) and Li foil discs (15 mm; MTI corporation); full cells were prepared with cathodes samples (14 mm) and capacity-matched graphite anodes (16 mm). Anodes were provided by the CAMP facility and contained 91.83 wt% SLC1520P graphite (Superior Graphite); 6 wt% PVDF binder (Kureha); 2 wt% C45 conductive carbon (Timcal); and 0.17 wt% oxalic acid; average full-cell *n*:*p* = 1.1:1). All cells were rested for 6 h at room temperature and were then cycled at 25°C using a multichannel cycler (Maccor). Cycling was conducted at a constant C/10 current (∼3 mA) with a 2 min rest period after each charge and discharge; half cells were cycled between 4.3 and 3.0 V while full cells were cycled between 4.2 and 3.0 V. Three replicate cells were prepared and tested at each condition.

Electrochemical impedance spectroscopy (EIS) was conducted after formation and after 25 C/10 cycles using a Verstat potentiostat/galvanostat (Princeton Applied Research). Half cells were analyzed at 3.65 V between 1 MHz and 100 mHz; full cells were analyzed at 3.55 V between 1 MHz and 10 mHz.

## 3 Results and discussion

### 3.1 Optimization of conditions for contaminant removal

#### 3.1.1 Optimized Al^0^ corrosion

The corrosion of Al^0^ under alkaline conditions proceeds according to a multi-step reaction, given in Eqs 1–4, resulting in a net consumption of 1 mole of OH^−^ per mole of Al^0^ and a net generation of 1.5x molar equivalent of H_2_ gas (Eq. 5) (Zhang et al., 2009):

$${Al}_{\left(s\right)}\to {Al}^{3+}+3e^{-}\left(anodic\right)$$ (1)

$$2H_{2}O+2e^{-}\to 2{OH}^{-}+H_{2\left(g\right)}\left(cathodic\right)$$ (2)

$${Al}_{\left(aq\right)}^{3+}+3{OH}_{\left(aq\right)}^{-}\to Al{\left(OH\right)}_{3\left(s\right)}$$ (3)

$$Al{\left(OH\right)}_{3\left(s\right)}+{OH}_{\left(aq\right)}^{-}\to Al{\left(OH\right)}_{4\left(aq\right)}^{-}$$ (4)

$${Al}_{\left(s\right)}+{OH}^{-}+3H_{2}O\to Al{\left(OH\right)}_{4\left(aq\right)}+3/2H_{2}$$ (5)

Thus, the rate and extent of Al^0^ corrosion may be evaluated through precise pH monitoring, with active alkaline Al^0^ corrosion indicated by a drop in the solution pH and reaction completion indicated by pH stabilization. Derivation of the association between solution pH and extent of Al^0^ corrosion is provided for reference in the Appendix (Supplementary Figure S2 and subsequent equations).

In the practical context of BM purification, complete and rapid corrosion of metallic contaminants is desired. Above pH 9, Al exists predominantly as the highly stable and highly soluble Al(OH)_4_^-^ product (K_f_ = 1.1×10^33^ at 25°C and solubility ∼0.97 g/L at pH 13°C and 25°C (Emamjomeh et al., 2011)). Given that complete corrosion of Al^0^ is thermodynamically predicted to occur at elevated pH, an emphasis was placed in the present work on identifying the process parameters leading to enhanced kinetics of the corrosion reaction—i.e., reaching full corrosion in a shorter time.

Previous work has indicated that corrosion rate is positively correlated with solution pH (Tabrizi et al., 1991; Aleksandrov et al., 2003; Reynolds et al., 2009; Zhang et al., 2009). Thus, further increasing solution pH is anticipated to further accelerate corrosion kinetics for metallic Al^0^. However, the combination of extremely alkaline solution and vigorous generation of H_2(g)_ poses significant safety concerns for practical adoption within an industrial direct recycling process. Thus, in the present study a solution pH of 13 (as measured at calibrated room temperature) was selected for all tests, such that the accelerating impact of additional process conditions could be evaluated.

**FIGURE 1:**
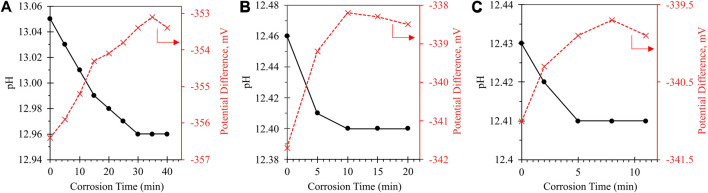
Impact of sonication on corrosion time of Al^0^: **(A)** Room temperature (20°C–22°C) with no sonication; **(B)** 40°C with no sonication; **(C)** 40°C with sonication. Solid curve with black circle markers refers to calibrated pH value (primary *y*-axis); dashed curve with red X markers refers to raw potential difference measured by pH electrode (secondary *y*-axis). Variations in the absolute value of the *y*-axes between samples are attributable to the temperature difference between calibration and testing conditions, as the pH meter was calibrated at room temperature prior to each round of testing.

Figure 1 shows the compounding effects of increasing sample temperature and introducing sonication on the corrosion kinetics of metallic Al. The variations in *y*-axis values between samples are associated with the temperature difference between calibration and testing conditions; specifically, the pH meter was consistently calibrated at room temperature prior to each round of testing, rather than at the sample temperature. Given that pH measurements are highly temperature-dependent, our calibration approach was found to improve sample reproducibility and ensure that all samples were prepared with the same nominal pH. However, as has been described and validated under constant-temperature conditions, the point at which the pH value stabilizes can be taken as an indicator of full Al^0^ corrosion.

As the sample temperature is increased from ∼20°C (uncontrolled room temperature) to a controlled 40°C, the time required for full ionization is reduced by 67% (30 min at room temperature to 10 min at 40°C). Introducing sonication at elevated temperature reduced the corrosion time by an additional 50%, enabling complete corrosion in 5 min. Both elevated temperature and sonication act to increase the molecular kinetic energy of the corroding system, increasing both the rate of surface reaction and the diffusion of solubilized Al(OH)_4_^-^ away from the corroding solid particle. Given that the dissolution of Al(OH)_3_ (solid surface layer) to Al(OH)_4_^-^ (soluble Al species; Eq. 4) is a purely chemical process, it is anticipated that corrosion kinetics will follow the two-step mechanism laid out by Balbaud-Célérier and Barbier—namely, dissolution will occur at the metal surface, followed by convective-diffusive transport away from the corroding particle into bulk solution (Balbaud-Célérier and Barbier, 2001). In this case, the bulk solution fluid properties significantly impact corrosion rate: At low fluid velocities, mass-transport will partially or entirely control corrosion rate; at higher flow rates, the laminar mass-transport layer becomes infinitely small, such that the corrosion rate is no longer mass-transport-limited and is instead activation-controlled (i.e., limited by the activation barrier of the corrosion reaction). Past a breakaway velocity point, the turbulence of the bulk flow can reportedly shear the surface layer off entirely (Zhang et al., 2009). We anticipate that the introduced combination of high-shear mixing and sonication is sufficient to induce this latter behavior, effectively targeting the rate-limiting step of Al^0^ corrosion (Pyun and Moon, 2000) by disrupting the formation of solid Al(OH)_3_.

The 5 min corrosion time achieved at 40°C with sonication was taken to be sufficiently rapid for practical contaminant ionization, and so higher-temperature systems were not studied in the present work; however, it is anticipated that further increasing the temperature would result in a further-reduced reaction time.

#### 3.1.2 Optimized Cu^0^ corrosion

As compared to metallic Al corrosion, the corrosion of Cu under alkaline conditions is significantly less favorable from both a thermodynamic (i.e., product solubility) and kinetic (i.e., reaction rate) perspective. In pure alkaline solution, corrosion products are limited to insoluble and passivating oxide species (CuO, Cu_2_O), which typically form at the outer surface of the corroding metallic particle and inhibit further corrosion. To achieve full ionization of metallic Cu in alkaline solution therefore requires the addition of a complexing agent, which binds free Cu^2+^ in solution more strongly than does free hydroxide. Previous work has utilized moderate-strength complexing agents such as ammonium hydroxide (NH_4_OH) (Tsang and Hailey, 2018). However, the use of stronger complexing agents (i.e., higher K value for metal-complexer bonding) with high solubility enables all newly ionized Cu^2+^ to be immediately and strongly bound in a soluble complex, thereby driving equilibrium towards further metal solubilization and increasing the overall extent of Cu corrosion. In this study, the chelating agent DTPA was introduced at >1 molar equivalent relative to Cu to ensure that the Cu would in all cases limit the total extent of reaction. The conditional stability constant log $K_{M^{\prime }L^{\prime }{\left(ML\right)}^{\prime }}=\frac{\left[{\left(ML\right)}^{\prime }\right]}{\left[M^{\prime }\right]\;\left[L^{\prime }\right]}$ = 16.4 for Cu/DTPA at pH 13 (M’ = Cu; L = DTPA; corrected for competing interactions of Cu and DTPA with OH^−^) (The Dow Chemical Company, 2021) implies extremely stable metal-ligand binding, such that the formation of Cu-DTPA is considered to be irreversible under the conditions studied. Further, the Cu-DTPA chelation reaction is rapid (typically assumed to be near-instantaneous), particularly relative to the reported rate of Cu corrosion (i_corr_ ≈ 2.98 × 10^–9^ A/cm^2^ under static mass transport conditions and a dissolved oxygen concentration of 8 ppm (King et al., 2010)). Thus, it is assumed that Cu-DTPA binding is not a rate-limiting reaction in the present tests.

**FIGURE 2:**
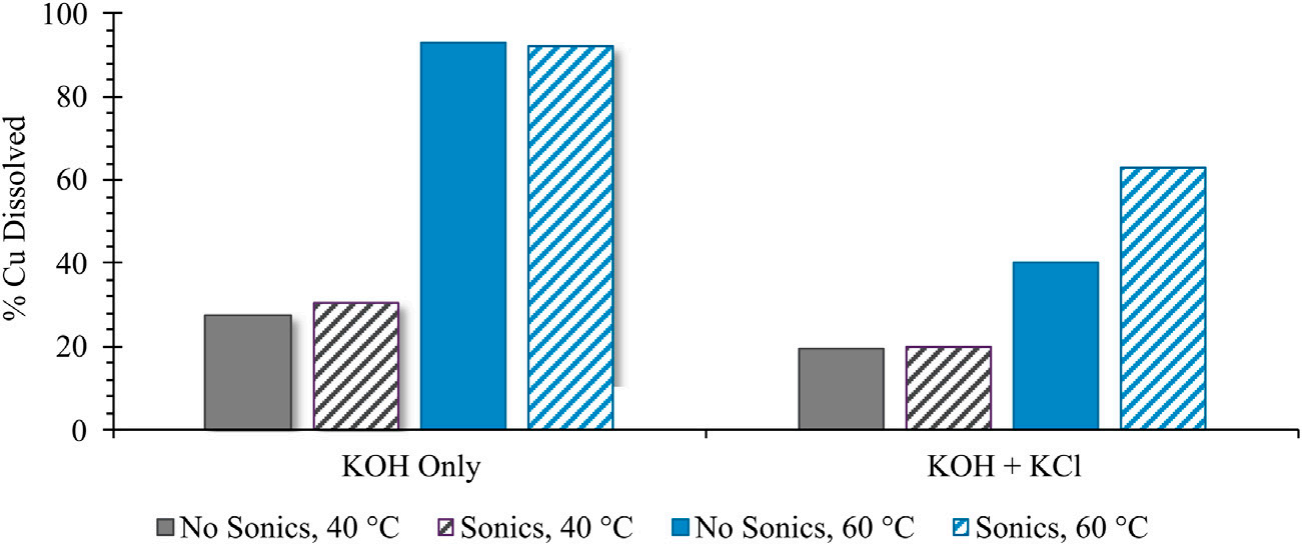
Impact of sonication, temperature, and presence of Cl^−^ salt on extent of Cu^0^ corrosion, reported as the percentage of initial Cu added dissolved in solution. All samples were analyzed using the reported colorimetric titration technique after 2 h of stirring time under ambient atmospheric oxygenation conditions.

**FIGURE 3:**
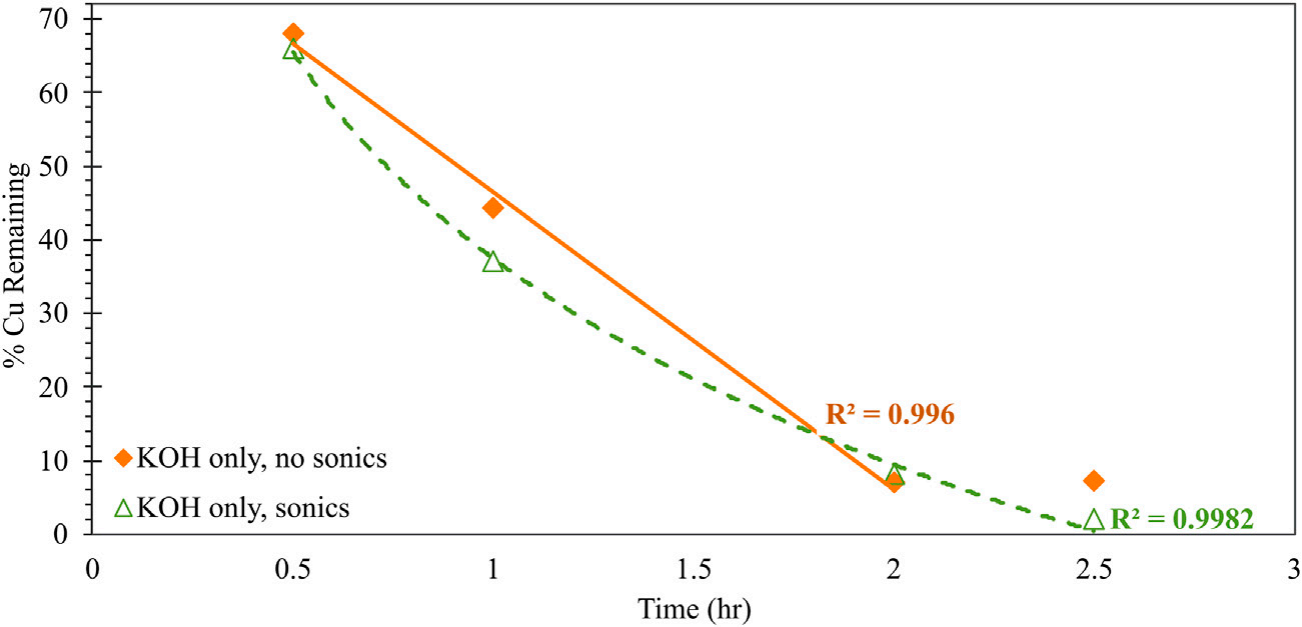
Impacts of sonication on the kinetic behavior of alkaline (0.1 M KOH) Cu^0^ corrosion, reported as the percentage of initial Cu added that is not bound to DTPA in solution. Condition of no sonication is indicated with solid orange diamonds; condition of sonication is indicated with open green triangles. Trendlines (solid for no sonication; dashed for sonication) are linear (*R* ^2^ = 0.996) and logarithmic (*R* ^2^ = 0.998) best-fits for the no-sonication and sonication conditions, respectively.

Figure 2 shows the kinetic impacts of a variety of process conditions on the total extent of Cu corrosion at a fixed time point (2 h); kinetic behavior under a subset of these conditions is reported in Figure 3. In both cases, quantification of Cu corrosion extent under varying process parameters was achieved *via* colorimetric titration, in which a known excess of DTPA was added and was back-titrated using Ca(NO_3_)_2_ salt in the presence of a colorimetric indicator. Quantification of remaining unbound DTPA in solution was used to calculate the corresponding amount of corroded Cu, bound in solution as Cu-DTPA, assuming the predicted 1:1 stoichiometry for the Cu-DTPA complex (Eivazihollagh, 2018). As shown in Figure 2, of the experimental variables analyzed, reaction temperature most strongly affects the extent of Cu^0^ corrosion at a fixed reaction time; this is particularly evident in the absence of Cl^−^ salt, where a nearly 2.4 times greater extent of corrosion is achieved at 60°C than at 40°C.

Based on a previous report suggesting that the lowest pitting potential for Cu occurs in a highly concentrated ∼5 M NaCl solution (Arjmand and Adriaens, 2012), KCl was added at an equivalent solution saturation (4.5 M) to evaluate the impacts on corrosion extent. Under such conditions, the presence of Cl^−^ was found to *reduce* corrosion extent relative to the equivalent system without Cl^−^; as indicated in Figure 2, this trend was independent of reaction temperature or the use of sonication. While such behavior belies the theorized instability of the Cu^0^ surface in the presence of Cl^−^ (Arjmand and Adriaens, 2012), it seems likely that operating under saturated Cl^−^ conditions—which concurrently increases solution viscosity—may either favor the formation of weakly bound surface-passivating species and/or severely reduce transport of ionized Cu away from the corroding particle. Such explanation is consistent with the data in Figure 2, which show that the use of sonication—i.e., increasing the local kinetic energy (i.e., convection) around the corroding particle and enhancing mass transport away from the corroding surface—has the greatest impact in enhancing corrosion for the Cl^−^-containing system at 60°C. The fact that sonication has less of an impact on corrosion enhancement for the Cl-containing system at 40°C suggests a temperature dependence of solution viscosity constraints on mass transport.

Our findings suggest that the mechanism of corrosion inhibition induced by Cl^−^ under highly alkaline conditions is primarily tied to surface or near-surface passivation, which sonication effectively disrupts. A detailed molecular reaction study to deconvolute these two contributions is beyond the scope of the present work, but the previous reports of King et al. support the notion of complex interactions of Cl^−^ with corroding Cu^0^ under highly alkaline conditions. Specifically, King et al. suggest that there is an initial competition between adsorbed (_ADS_) OH^−^ and Cl^−^ ions for surface sites on the corroding Cu (Eqs 6–8):

$$Cu+2{OH}^{-}⇌Cu{\left(OH\right)}_{2ADS}+e^{-}$$ (6)

$$Cu+{Cl}^{-}⇌{CuCl}_{ADS}+e^{-}$$ (7)

$${CuCl}_{ADS}+2{OH}^{-}⇌Cu{\left(OH\right)}_{2ADS}+Cl$$ (8)

Systems with higher concentrations of Cl^−^ and higher rates of mass transport typically favor the subsequent formation of CuCl_2_^−^, as per Eq. 9:

$${CuCl}_{ADS}+{Cl}^{-}⇌{CuCl}_{2}^{-}\left(surface\right)\to {CuCl}_{2}^{-}\left(bulk\right)$$ (9)

Conversely, at higher pH (as in the present system), CuO formation is favored; in particular, at pH 13, Eq. 10 is expected to predominate the anodic corrosion reaction:

$$Cu{\left(OH\right)}_{2\;ADS}⇌Cu{\left(OH\right)}_{2}\left(surface\right)⇌CuO\left(surface\right)+H_{2}O$$ (10)

In the system of study—which contains both highly concentrated Cl^–^ and high pH—it is not readily apparent from theory whether Eq. 9 or Eq. 10 should dominate, and it is likely that an equilibrium exists between both. However, in the current study, the observed surface passivation (i.e., reduced corrosion extent) in the presence of concentrated Cl^−^ strongly suggests that mass transport is restricted—ostensibly due to high solution viscosity—which would hinder the rate-limiting step of CuCl_2_^−^ diffusion from surface to bulk (Eq. 9) and increase the favorability of passivating CuO evolution.

Finally, the contribution of the chelating agent DTPA to overall corrosion behavior must be considered. Given the extreme steric bulk of DTPA, it is presumed that active surface reaction occurs primarily between Cu and the small anionic species (OH^−^, Cl^−^) and Cu-DTPA binding (i.e., displacement of OH^−^ by DTPA^−^) proceeds only after the relevant surface species (CuOH, CuCl_2_^−^) have diffused to the bulk. Thus, the restricted mass transport around the corroding Cu particle induced by high solution viscosity in concentrated Cl^−^ solution limits accessibility to DTPA binding sites and therefore reduces overall solubilized Cu.

In the present work, all tests involving Cl^−^ were conducted under saturated conditions. However, it is likely that an optimum concentration of Cl^−^ below saturation may exist where corrosion-enhancing tendencies (i.e., reduction of pitting potential and destabilization of the corroding surface layer) are not counteracted by corrosion-suppressing tendencies (i.e., favoring the formation of Cu_2_O and restricting near-surface transport). Ongoing studies are underway to evaluate varying Cl^−^ concentration on overall corrosion extent and further optimize solution composition to accelerate Cu^0^ corrosion.

Figure 3 further explores the kinetic benefit of sonication on Cu^0^ corrosion. Here, the residual amount of Cu remaining in solution - as measured via colorimetric back-titration - is tracked as a function of reaction time, for equivalent systems either subject to only overhead stirring or to concurrent stirring and sonication. In the absence of Cl^−^, sonication appears to have a negligible impact on the overall extent of corrosion at the fixed timepoint (2 h reaction time) shown in Figure 2. However, the time-dependent behavior in Figure 3 reveals that the sonication does, in fact, induce a shift in the kinetic mechanism of corrosion. Specifically, Cu^0^ corrosion proceeds *via* zeroth-order kinetics (evidenced by the linear relationship between concentration and time; *R* ^2^ = 0.996) for the system without sonication; conversely, Cu^0^ proceeds *via* first-order kinetics (logarithmic relationship between concentration and time; *R* ^2^ = 0.998) when sonication is employed.

Zeroth-order kinetics‐here, the no-sonication condition‐implies no dependence of reactant or product species on overall reaction rate. In the present system, this represents the corrosion of Cu^0^ to Cu^2+^, which—due to the high binding stability constant K for the Cu/DTPA complex and excess DTPA reagent—is expected to rapidly and irreversibly bind to free DTPA in bulk solution. The first-order kinetics associated with the sonication condition imply a first-order dependence of overall reaction rate on the concentration of reacting species. Sonication introduces concentrated kinetic energy in the near vicinity of the corroding Cu^0^ particle—which is anticipated to accelerate the ionization of Cu^0^ to Cu^2+^ at the particle surface—and also induces convective transport around the particle surface. The observed first-order kinetics implies that this surface reaction is occurring faster than the ionized Cu^2+^ can be transported away from the corroding particle into the bulk solution, where binding to the sterically hindered DTPA ligand can occur. Such restricted transport will induce a local concentration gradient in the near-surface region of the Cu^0^, limiting the rate of further corrosion. The observed behavior highlights the critical contribution of mass transport to both the rate and extent of Cu corrosion under alkaline conditions, and also strengthens the earlier-described mechanism for Cl^−^-induced corrosion inhibition. Finally, it is notable that the overall extent of corrosion plateaus at ∼93% after 2 h in the absence of sonication, whereas full corrosion (within the 2% error bounds of the colorimetric quantification method) is achieved after 2.5 h with sonication. This may indicate the formation of surface-passivating Cu_2_O in the former system, which sonication may be sufficient to disrupt in the latter.

### 3.2 Evaluating BM purification conditions on NMC

Optimal conditions for the purification of black mass must both simultaneously maximize the corrosion rate and extent of the undesired contaminants (Al^0^, Cu^0^) and minimize adverse impacts to the physico-electro-chemical performance of the target cathode active material. It should be emphasized that, in the context of practical implementation in a direct recycling line, the “no-treatment” condition is the worst outcome: as noted in Section 1 and highlighted in Section 3.2.2, the fine fraction of the black mass is unviable for direct recycling when even low levels of Al^0^ and Cu^0^ contamination are present. However, the ultimate target for a successful BM purification technique is to induce no measurable effect on the active NMC. It is therefore imperative to evaluate the effects of treatment on a representative cathode material to identify disparities between pristine and treated material, and to iteratively improve upon treatment solution chemistry and associated process conditions.

In the present work, a sample of pristine NMC-111 was exposed to the process conditions tailored for the corrosion of Al^0^ and Cu^0^, as described in Section 3.1. Testing these treatment conditions on NMC revealed several relevant parameters for optimum BM purification that were not evident from the idealized corrosion studies alone. For example, while the counter-cation for OH^−^ and Cl^−^ salts does not significantly impact the corrosion behavior of Al^0^ or Cu^0^ in alkaline solution, it does critically influence cation exchange tendency in NMC. Treating NMC with NaOH and NaCl results in significantly reduced full-cell capacity relative to pristine NMC-111 (Supplementary Figure S3), which we attribute to a loss of cyclable Li^+^ due to Na^+^/Li^+^ exchange, enabled by the similar ionic radii of the two ions (1.02 Å for Na^+^, *vs*. 0.76 Å for Li^+^). Capacity in treated NMC was significantly improved by switching to K^+^ salts, where the larger ionic radius of K^+^ (1.38 Å) effectively precludes the possibility of K^+^/Li^+^ exchange.

Informed by both the results presented in Section 3.1 and the additional iterative optimization described above, solution and process conditions were selected for testing on pristine NMC-111 to demonstrate no adverse impact to the target cathode material. In particular, NMC-111 was exposed to solution at pH 13 (nominal 0.1 M KOH, with or without the presence of concentrated Cl^−^) and was stirred under ambient oxygen at 60°C for 2.5 h.

#### 3.2.1 Impacts of BM purification conditions on pristine NMC-111

The reported black mass purification treatment conditions do not induce detectable changes to the bulk structural or morphological properties of NMC. As shown in Supplementary Figure S4, scanning electron microscopy (SEM) suggests that the purification treatment retains the secondary particle (“meatball”) structure of the NMC-111, with no observed particle fracture. Further, bulk powder XRD indicates that there is no significant difference in lattice parameters or peak intensity ratios between treated and pristine NMC (Supplementary Figure S5; Supplementary Table SA1).

**FIGURE 4:**
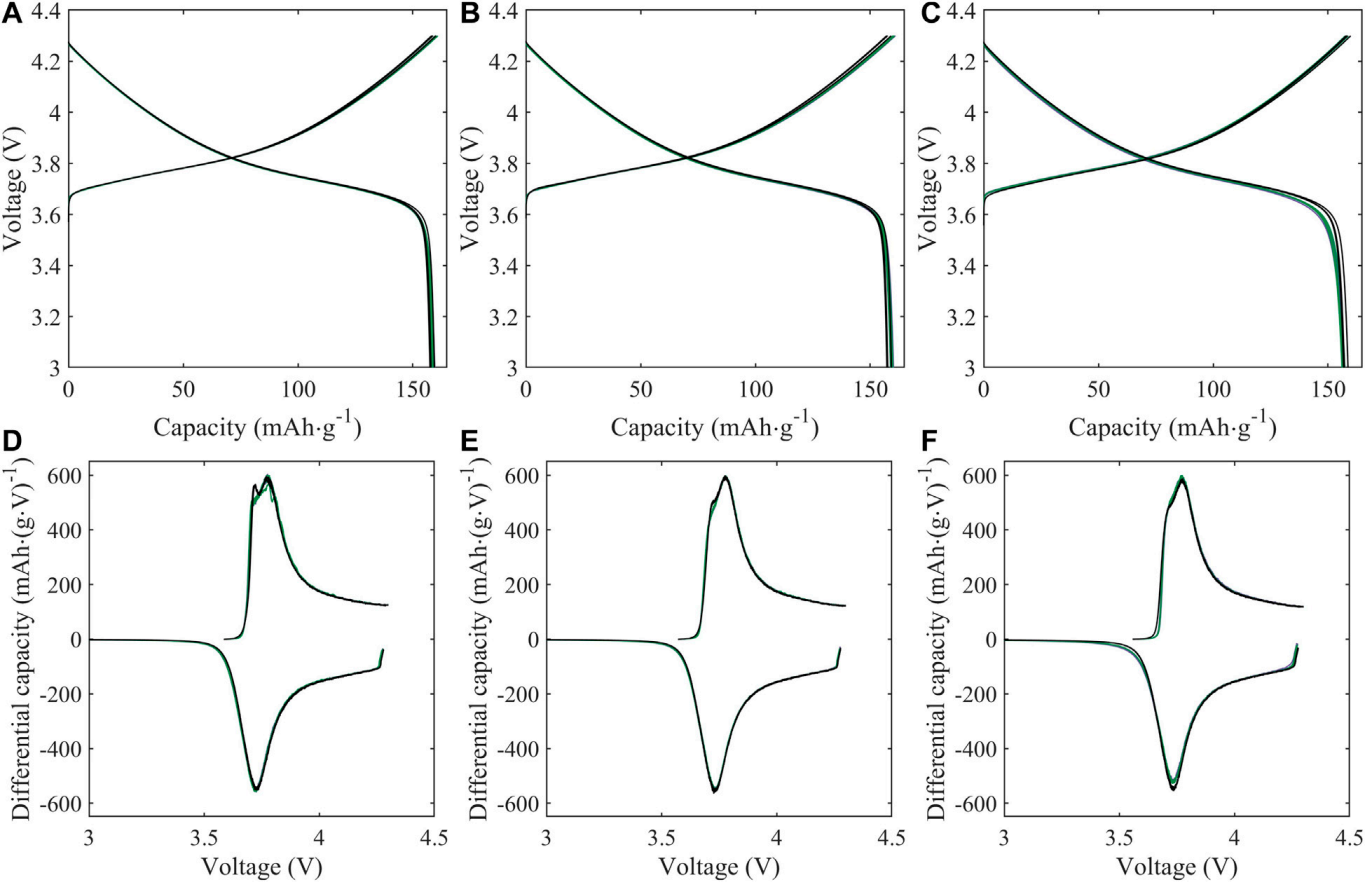
Voltage profiles **(A–C)** and differential capacity (dQ/dV; **(D–F)**) of half cells prepared with NMC-111: pristine (black) or following treatment in KOH (green) or KOH + KCl (purple) matrix. Data is reported for the initial formation cycle **(A,D)**; the final formation cycle **(B,E)**; and the 10th C/10 cycle following formation **(C,F)**. For plots **(A**–**C)**, cell replicates are shown as multiple curves of the same color; for plots **(D**–**F)**, one representative cell is shown for each condition. All treated NMC samples were subject to the same processing environment (i.e., sonication at 60°C for 2.5 h).

**FIGURE 5:**
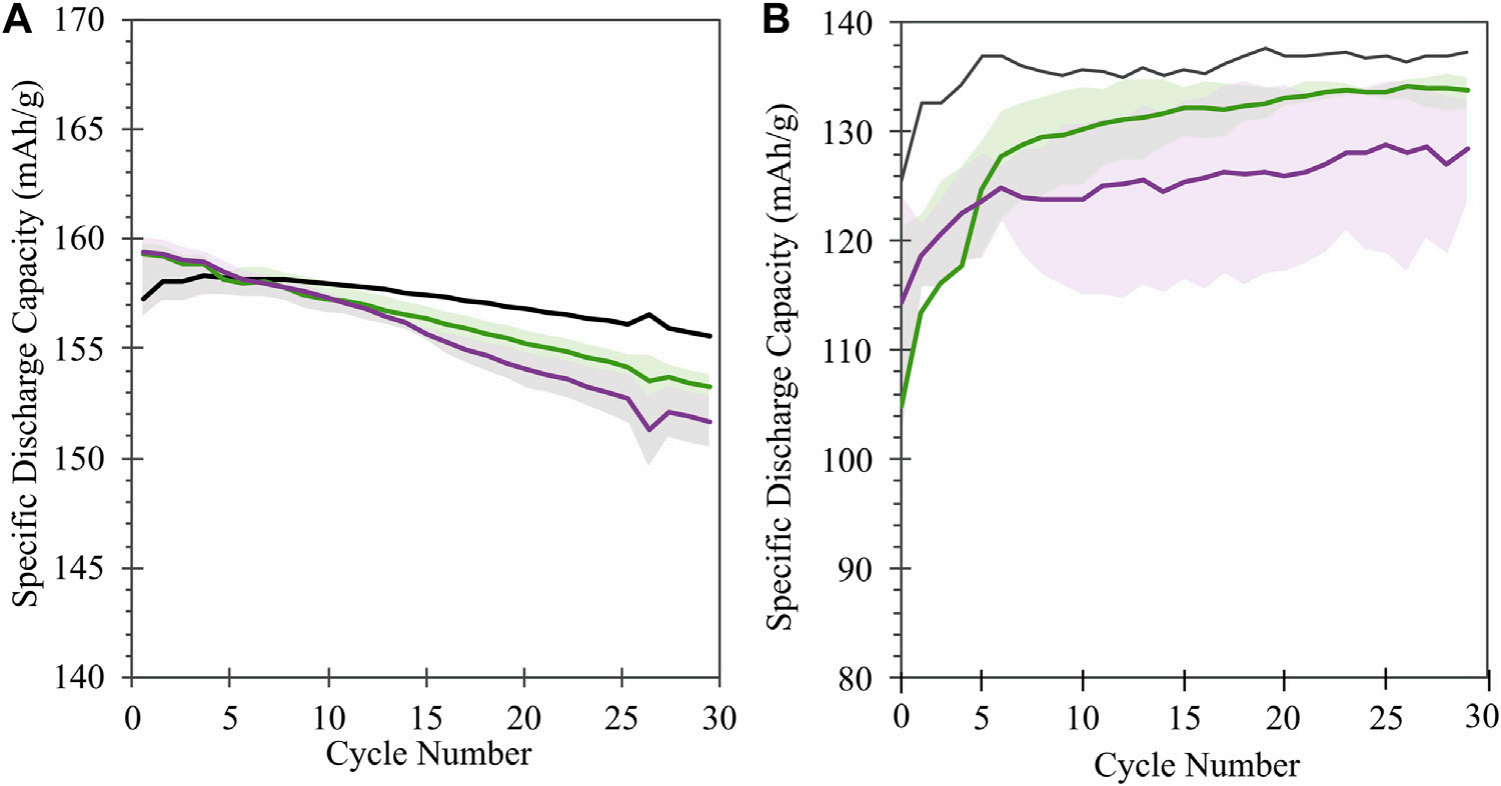
**(A)** Half cell and **(B)** full cell cycling performance of NMC samples: pristine (black) or following treatment in KOH (green) or KOH + KCl (purple) matrix. Shaded regions reflect the range of performance from equivalent sample replicates for each condition; solid lines reflect the average value across replicates.

Half cell electrochemical capacity is not significantly impacted by the treatment conditions, with voltage profiles of treated material matching that of pristine material over the course of initial cycling (Figure 4) and cycling stability comparable (within 5 mAh/g) between treated and untreated material over initial C/10 cycling (Figure 5A). For both treatment conditions (KOH and KOH/KCl), a higher overpotential upon initial charge is observed in the first cycle, followed by a drop in voltage (Figure 4A). This erratic initial behavior is more clearly evident in plots of differential capacity (Figure 4D), which indicate a shift to higher potential of the initial oxidation peak; this is consistent with higher overpotential at the beginning of charge. Further, significant noise in the differential data is observed between 3.8 and 3.85 V. Given that this irregularity is rapidly resolved—with the performance of the treated material becoming indistinguishable from that of the pristine halfway through the first charge (90 mAh/g capacity)—it may reasonably be concluded that such behavior is tied to the presence of a finite degree of chemical or structural surface impurity due to treatment, which disrupts initial surface film formation (SEI/CEI) and is subsequently consumed or passivated. In half cells, there is no noticeable difference in either voltage profiles (Figures 4B, C) or differential capacity behavior (Figures 4E, F) between pristine and treated cells after the initial charge step.

**FIGURE 6:**
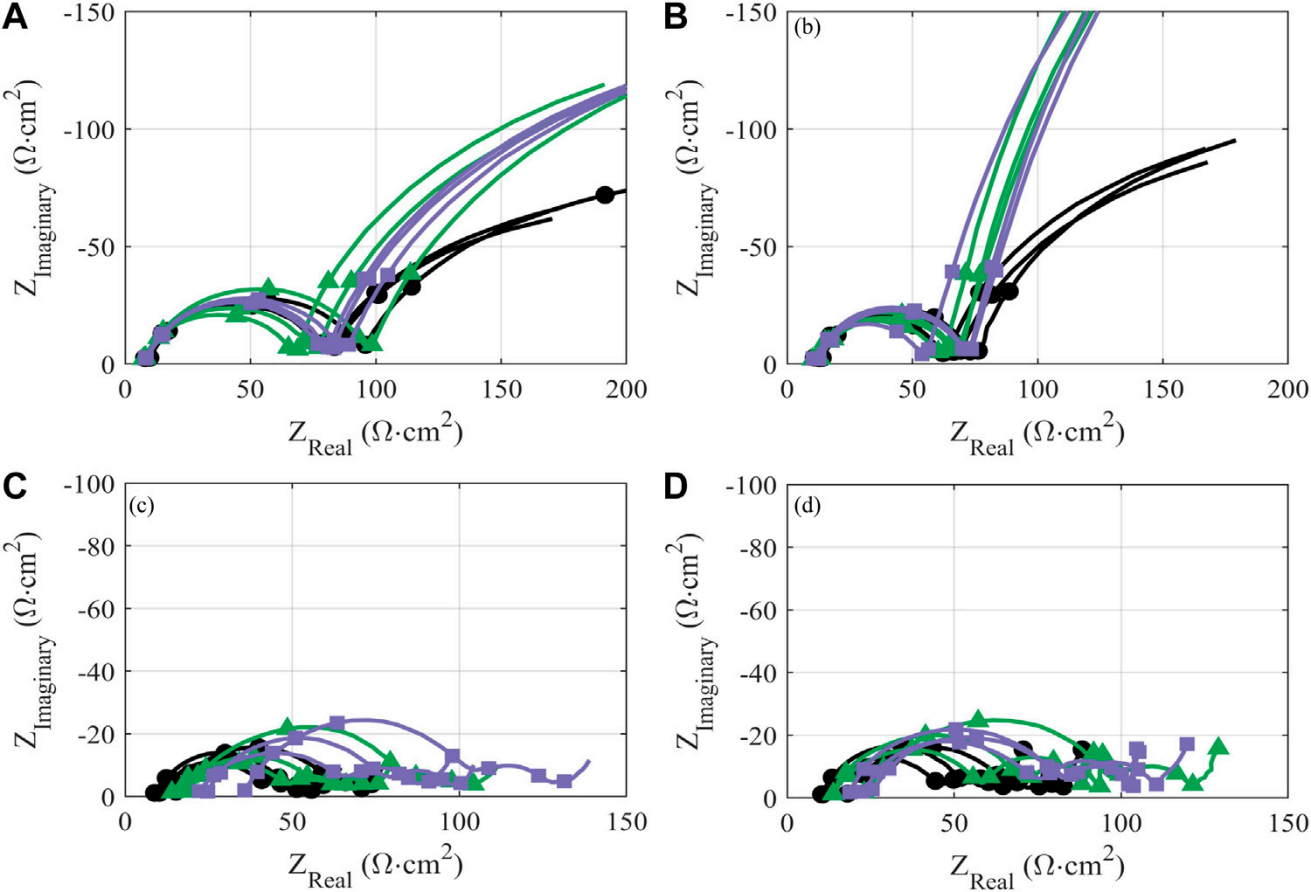
Area-normalized impedance of half cells **(A,B)** and full cells **(C,D)** prepared with NMC-111: pristine (black circles) or following treatment in KOH (green triangles) or KOH + KCl (purple squares) matrix. Measurements are reported after formation cycles **(A,C)** and after 25 cycles at C/10 following formation **(B,D)**. All treated NMC samples were subject to the same processing conditions (i.e., sonication at 60°C for 2.5 h). The markers on the impedance graphs are located on the frequency decades, starting at 10^5^ Hz on the lower left-hand side of the axis and at every frequency decade until the end of the measurement.

Impedance growth in half cells over the first 25 cycles is also comparable between treated and pristine material, within the range of cell-to-cell variability (Figures 6A, B). The reduction in charge-transfer resistance over initial cycling for all cells—indicated by the reduced diameter of the primary semicircle—is consistent with previously evaluations of polycrystalline cathode materials (Safari and Delacourt, 2011; Yari et al., 2021). Such impedance response implies improved charge-transfer kinetics upon initial cycling; this is typically attributed to increased exposure of the active material to electrolyte due to decrepitation (i.e., microcracking) between primary particles, increasing the overall active surface area (Christensen, 2010; Trevisanello et al., 2021).

**FIGURE 7:**
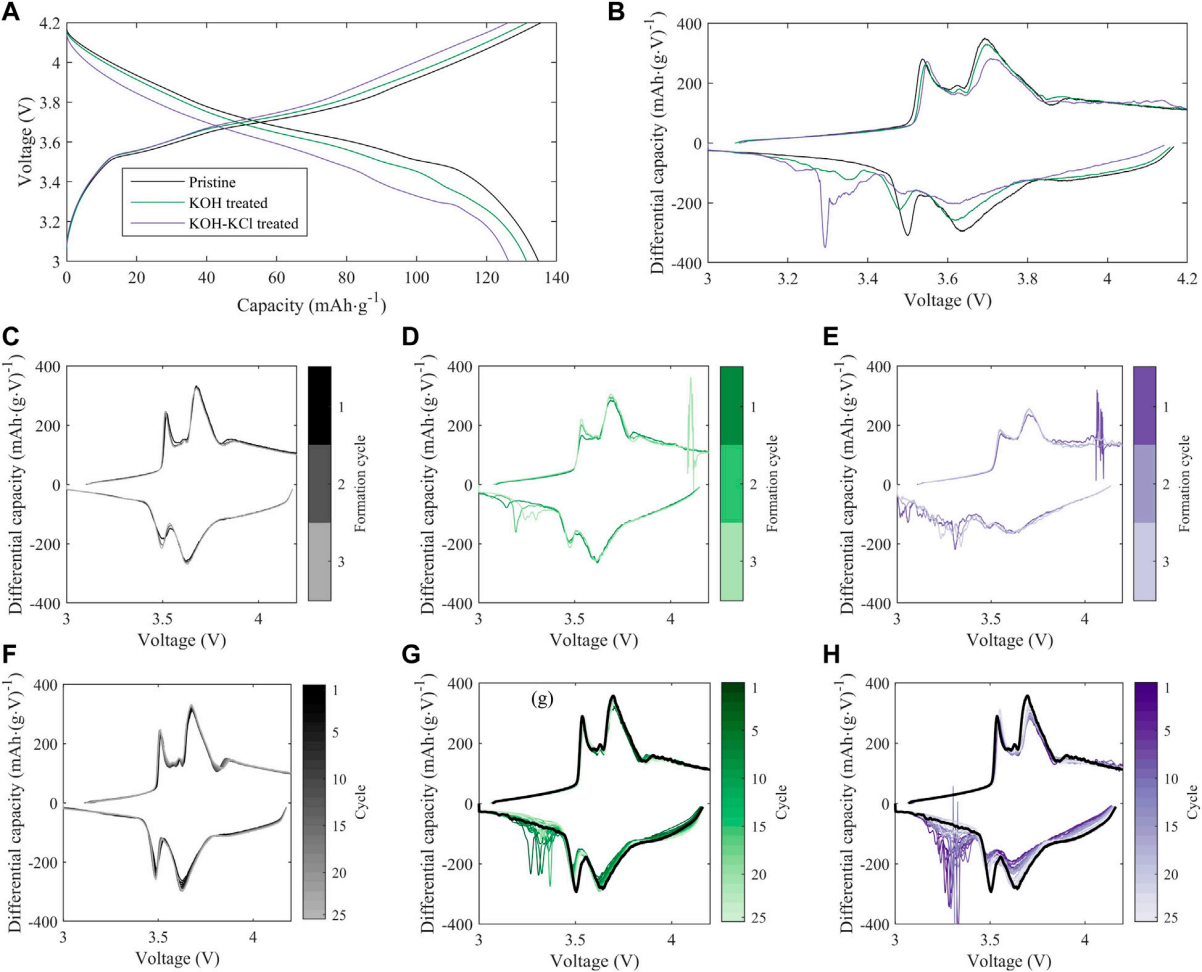
Evolution of full-cell electrochemistry with cycling for treated vs. pristine NMC-111: **(A)** Full-cell voltage profiles at the 10th C/10 cycle following formation; **(B)** Differential capacity (dQ/dV) profiles for the cells and cycle in **(A)**; **(C–E)**: Evolution of differential capacity during formation cycling for **(C)** pristine, **(D)** KOH-treated, and **(E)** KOH + KCl-treated cells; **(F–H)** Evolution of differential capacity during 25 subsequent C/10 cycles for **(F)** pristine, **(G)** KOH-treated, and **(H)** KOH + KCl-treated cells. For **(C–H)**, line colors indicate the cycle number, increasing from dark to light tones. In **(G,H)**, black overlay indicates the first post-formation cycle of the pristine cell in **(F)**. In all sub-plots, data from a single representative cell replicate is shown.

In full cells (i.e., NMC vs. graphite), a greater disparity is observed between the cycling performance of treated and pristine materials, particularly during initial formation (Figure 5B), with both KOH- and KOH/KCl-treated materials showing reduced capacity relative to pristine. Interestingly, the capacities of treated materials recover somewhat with repeated C/10 cycling, but the full value of pristine capacity is not achieved after the initial 30 cycles reported. Previous studies on refurbished cathode materials processed in aqueous solution matrices reveal a slight capacity reduction relative to pristine material (Folayan et al., 2021; Xu et al., 2021); however, the majority of electrochemical analysis to date on directly recycled material is reported only for half-cell configuration. Thus, to our knowledge, a thorough explanation for the observed disparity between half cell and full cell behavior in these materials has yet to be put forward, and is the subject of our continuing investigation. Differential capacity analysis (Figure 7) reveals a significant disruption to expected electrochemical behavior for the treated material during formation, and implies an adverse impact of the Cl-containing treatment conditions on the subsequent performance of the NMC. Several novel oxidative peaks are observed near the top of charge (>3.8 V) for cells treated under both conditions. For cells treated with only KOH, this high-voltage oxidative instability appears to be largely passivated after the first cycle; however, for cells treated with KOH + KCl, such behavior continues throughout formation. KOH/KCl-treated cells also show highly erratic behavior upon reduction, particularly below 3.4 V.

**FIGURE 8:**
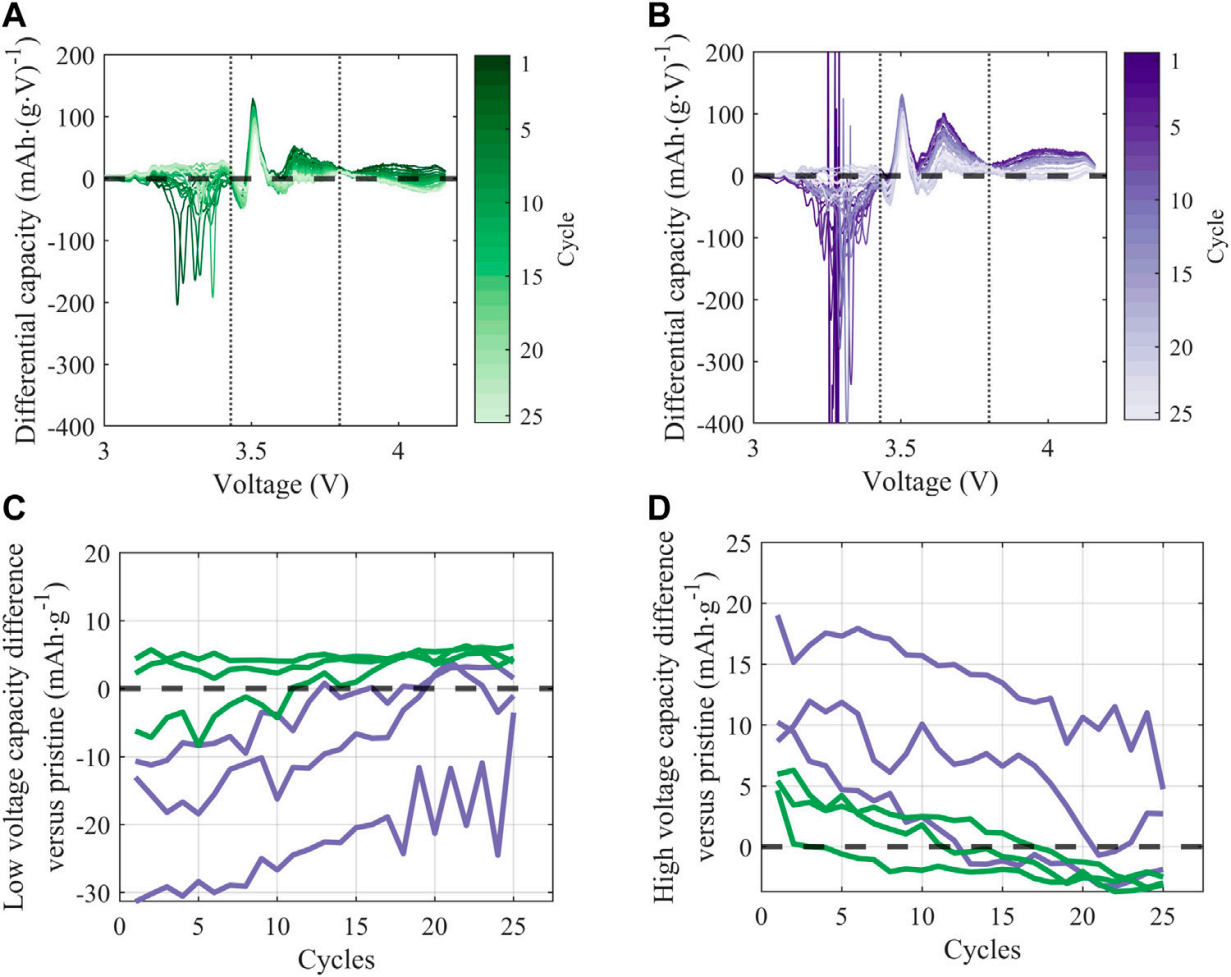
**(A,B)** Differential discharge capacity over 25 C/10 cycles (following formation) for a representative cell treated with **(A)** KOH and **(B)** KOH/KCl; vertical dashed lines at 3.43 V and 3.8 V denote the low, middle, and high voltage regions. Normalized dQ/dV is reported as the value from the treated cell minus the first-cycle value from a representative pristine cell **(C,D)** Capacity from side reactions in **(C)** low-voltage (3–3.43 V) and **(D)** high-voltage (3.8–4.2 V) regions, calculated as the integrated signal area. Lines of the same color reflect cell replicates of the same treatment condition (KOH, green; KOH/KCl, purple).

Further, there is evidence for ongoing reactivity in full-cells prepared with treated material with repeated electrochemical cycling. In Figure 8, the additional reactivity observed upon discharge in the full cells prepared with treated NMC is quantified by normalizing differential capacity (dQ/dV) with respect to the pristine material. Normalization is achieved by subtracting the first-cycle post-formation dQ/dV intensity of a representative pristine cell from the dQ/dV intensity of each treated cell. Data for a representative cell of each treatment condition (KOH and KOH/KCl) is shown in Figures 8A, B. Normalized dQ/dV curves show that the treated material exhibits irregular behavior in the regions outside of the window of predominant graphite (∼3.5 V) and NMC (∼3.7 V) redox, indicated by vertical dashed lines. In the low-voltage (<3.43 V) region, curves deviating in the negative direction from the baseline (horizontal dashed black line) implies increased reductive reactivity upon discharge. Such excess capacity can be attributed to adverse side reactions, and corresponds to a non-negligible portion of overall observed capacity. For example, at the 5th C/10 cycle post-formation, extraneous capacity observed for the KOH/KCl-treated material in the low-voltage region accounts for ∼14.5% of total capacity (taken as an average across cell replicates). The material treated with KOH only shows relatively less low-voltage reactivity, with some variations between samples.

Behavior of the treated material in the high-voltage (>3.8 V) is graphically inverse to that in the low-voltage region: i.e., curves deviating in the positive direction from the baseline are taken to represent worse performance. The high-voltage region, as delineated, is outside of the “peak” regime of NMC redox but is still associated with reversible NMC electrochemical activity, and thus normalized capacity values lying above the baseline imply reduced reductive capacity upon discharge. The reduction in capacity observed in this high-voltage region for treated material—particularly KOH/KCl-treated material—suggests increased polarization and reduced electrochemical accessibility of the NMC, which is likely the result of a resistive residual surface film. This interpretation is consistent with the increased resistance observed for treated materials (Figures 6C, D).

For all treated cells, irregular behavior in the low-voltage regime is found to diminish with continued cycling, such that normalized differential capacity curves of the treated material trends towards that of the baseline. Interestingly, across all replicates of treated material, differential capacity intensity in both the primary NMC redox region (3.43–3.8 V) and higher-voltage reversible redox region (>3.8 V) show a continuous *increase* with repeated cycling. This can be observed directly (i.e., increase in dQ/dV intensity) in Figures 7G, H, and in inverse (i.e., decline of positive normalized dQ/dV values) in Figures 8A, B. Such behavior is in contrast to the pristine material, which shows stable or slightly declining peak intensity with cycling in the mid-voltage region (Figure 7F).

Taken together, differential capacity behavior implies the presence of reactive contaminant(s) in the treated material, which is electrochemically consumed during cycling. Such species appear to initially prevent effective utilization of the Li inventory in the NMC, but do not necessarily deplete significant Li through reaction: as shown in both Figure 5B; Figure 8, the capacity and differential capacity behavior of treated material approaches that of pristine material upon continued cycling (i.e., as the reactive contaminant(s) are consumed). Notably, significantly higher reactivity is observed in full cells (NMC vs. graphite) than in half cells (NMC *vs*. Li), suggesting a strong role of the graphite anode in facilitating electrochemical side reactions.

The observed novel reactivity in full cells subject to BM purification treatment conditions is anticipated to arise from two primary sources: 1) The electrochemical reaction of residual chemical species, i.e., from the treatment solution, with the counter electrode; 2) Surface-structural rearrangement of the NMC induced by the treatment conditions; previous reports suggest that these two processes are often linked. Specifically, the reactivity of NMC in aqueous solutions is a strong function of pH, with mechanisms of Li^+^/H^+^ exchange tied to the availability of free H^+^ in solution. In neutral or acidic environments, Li^+^/H^+^ exchange and subsequent reactivity is reported to occur according to the following pathway (Eqs 11–13) (Wood et al., 2020):

$${Li}^{+}+H_{2}O⇌LiOH+H^{+}$$ (11)

$$LiOH+{CO}_{2}⇌{LiHCO}_{3}$$ (12)

$$LiOH+{LiHCO}_{3}+{CO}_{2}⇌H_{2}O+{Li}_{2}{CO}_{3}$$ (13)

The previously noted (Tsang and Hailey, 2018) and herein confirmed stability of NMC in highly alkaline aqueous solution can be attributed to the suppression of the above Li^+^/H^+^ exchange pathway in the absence of either free H^+^ or other Brønsted–Lowry acids. Thus, while the treatment conditions themselves are inert to NMC, the post-treatment protocol employed for these samples involves a brief rinse with deionized water (pH 7). The short timescale of the rinse is insufficient to induce major bulk Li^+^/H^+^ exchange, as verified through a lack of bulk structural rearrangement (Supplementary Figure S5; Supplementary Table SA1) which would be associated with Li leaching. However, this rinse may be sufficient to induce such reactivity at the NMC surface, and could therefore reasonably produce LiOH, LiHCO_3_, and Li_2_CO_3_ at the NMC surface. The formation of these surface contaminant species is typically associated with detrimental performance in high-Ni cathodes (Pritzl et al., 2019; Martinez et al., 2020), where they are observed to evolve even with ambient exposure to atmosphere (Martinez et al., 2020), but may also be formed in the present system of NMC-111 due to direct exposure with deionized water in a high volume concentration (i.e., ∼30:1 DI H_2_O:NMC-111 during the post-treatment rinse). The presence of such surface species could account for the elevated charge-transfer resistance observed in full-cells for both sets of treated material (Figures 6C, D). Additionally, the electrochemical oxidation of residual surface LiOH is expected to occur at ∼3.2 V *vs*. graphite, which may contribute to the erratic low-voltage differential capacity behavior observed for treated cells with continuous cycling (Figures 7D, E, G, H).

Further, the migration of Li during such surface leaching is anticipated to result in a near-surface structural transformation to a Li-containing spinel and/or rock-salt phase (Martinez et al., 2020). While it is difficult to quantify low phase percentages in bulk powder XRD measurements, Rietveld refinement may be used to calculate total phase impurity, including LiMn_2_O_4_-type spinel, M_3_O_4_-type spinel (e.g., Co_3_O_4_), and NiO-type rocksalt—all of which have been reported to result from phase transformation of NMC (Bak et al., 2014). A total phase impurity of 8.2% is observed in both the KOH-treated and KOH/KCl-treated samples (Supplementary Table SA1), as compared to 7% in the baseline (untreated) sample, which supports the notion of surface-structural rearrangement. Further, the presence of such surface phase impurity has been implicated in the growth of charge transfer resistance on high-Ni cathodes (Pritzl et al., 2019); this is supported by the present data, which shows a growth in charge transfer resistance in the full cells prepared with cathode material undergoing treatment and subsequent rinsing (Figures 6C, D). However, the presence of such surface phase impurity due to post-treatment washing cannot fully account for the anomalies in observed performance between half and full cells. The novel reduction peaks observed in differential capacity analysis (dQ/dV) for full cells do not align with the expected reversible redox peaks for either LiMn_2_O_4_-type or M_3_O_4_-type spinel (Thackeray, 1997; Bruce et al., 1999; Teufl et al., 2018), although the cycling parameters employed in this study may not have been sufficient to activate the predominant spinel oxidation peak (which is reported to occur at ∼4.5 V *vs*. Li^+^/Li in a mixed NMC/Li_2_MnO_3_ system) (Teufl et al., 2018).

Finally, we anticipate that residual K^+^ and Cl^−^ present at the cathode may also migrate to the anode during electrochemical cycling. Xu et al., 2021. have evaluated delithiated NMC-111 processed in concentrated KOH solution *via* XPS, and have detected peaks related to K 2p_3/2_ and K 2p_1/2_—suggesting some degree of K^+^ intercalation to the NMC lattice in the near-surface region (Xu et al., 2021). Such intercalation must necessarily be minor in the present study, as no impact to bulk lattice properties were observed. However, we anticipate that this minor intercalated species—along with any residual K^+^ salts (e.g., KOH) remaining on the surface—may solubilize in the electrolyte and facilitate adverse reaction pathways at the anode. The reversible (de) intercalation of K^+^ into graphite has been reported at 0.1/0.32 V *vs*. Li/Li^+^ (Stage 1) with phase transitions to Stage 2 and Stage 3 reported at 0.23/0.41 V and 0.34/0.52 V *vs*. Li/Li^+^, respectively (Zhao et al., 2016). Thus, it is plausible that K^+^ intercalation may contribute to the extraneous reactivity observed when the anode is in a highly reduced state, i.e., top of charge for the full cell. Further, the intercalation of anions into graphite may occur during full-cell discharge; in particular, the insertion and limited de-insertion of Cl^−^ and Cl^−^-containing complex anions into graphite has been reported under oxidizing conditions (Zhang and Lerner, 1993; Guo et al., 2021), which occur at the bottom of discharge in the full cell. Since the present system does not contain an electrolyte and cell conditions optimized for reversible reaction of alternative ions, we anticipate that any such intercalation would be irreversible and would consume the reactive species. The electrochemical reactivity of free K^+^ and/or Cl^−^ remaining in solution could also directly disrupt SEI formation through the formation of complex solids, for example through Li-Cl interactions or through K^+^/Li^+^ substitution in SEI products. Such complex surface species may also contribute to increased full-cell resistance, relative to the pristine material (Figure 6C, D).

Detailed deconvolution of surface reactions and structural rearrangement leading to the observed irregularities in full cells is beyond the scope of the present effort, but is the focus of continuing work and will be the subject of a subsequent publication. It should be noted that the observed surface impurities leading to somewhat suppressed full cell performance are considered a minor impact relative to the detrimental impact of the Al and Cu contaminations which the reported BM purification addresses, as will be discussed in the following section.

#### 3.2.2 Bulk physical and electrochemical impacts of treatment on simulated BM

Following preliminary evaluation of treatment conditions on pristine NMC-111, a “simulated BM” was prepared to more closely reflect a practical BM feedstock while minimizing the additional sample matrix complexity in BM. Specifically, pristine NMC-111 was spiked with 1 wt% of metallic contaminant (Al or Cu) and was subjected to optimized treatment conditions, as outlined in Section 3.2.1. Cu-spiked samples also included 2x molar DTPA, relative to the concentration of Cu^0^. As mentioned, the metallic impurity concentration selected (1 wt%) is consistent with contamination levels observed in the fine fraction of industrial shredded BM (Fink et al., 2022), and is thus is a relevant metric to evaluate the present methods for industrial application.

**FIGURE 9:**
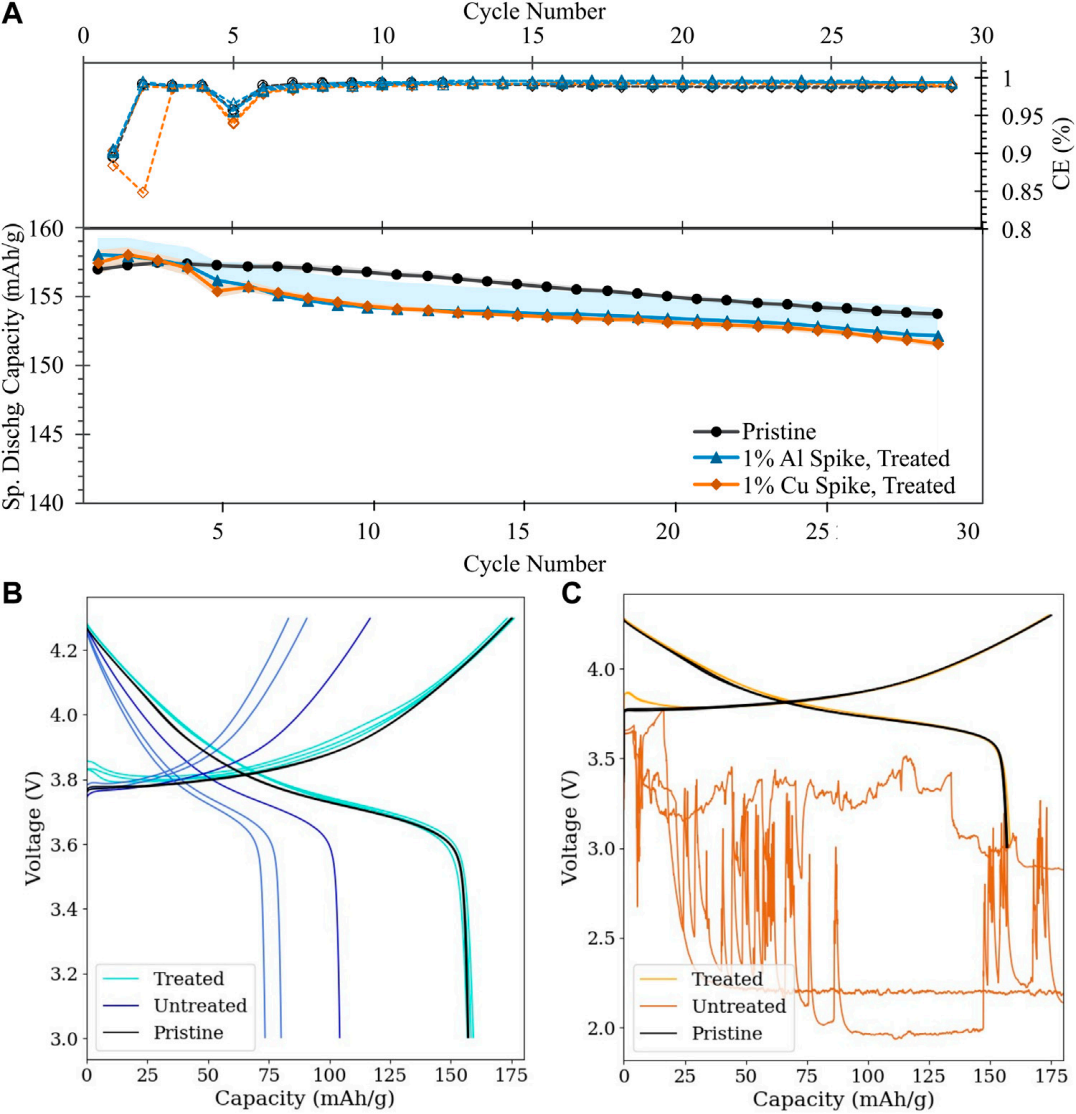
Half-cell cycling performance of simulated BM: **(A)** Cycling stability and Coulombic efficiency of treated simulated BM (Al contamination: blue triangles; Cu contamination: orange diamonds) *versus* pristine NMC-111 (black circles); **(B,C)** First-cycle voltage profiles of treated *vs*. untreated simulated BM **(B)**: Al contamination; **(C)** Cu contamination; darker tone indicates untreated condition. Lines of the same color reflect cell replicates.

As shown in Figure 9A, the optimized treatment conditions are sufficient to recover the half-cell electrochemical capacity of the pristine material, within several mAh/g. The improvement with treatment is particularly striking when compared to the electrochemical behavior of the untreated simulated BM (Figures 9B, C and Supplementary Figure S5), which imply that contamination levels as low as 0.1 wt% for both metals are sufficient to significantly disrupt voltage behavior and contamination at 1% or above induces catastrophic cell failure in Cu^0^-contaminated cells. Such capacity recovery in highly contaminated cells demonstrates initial validation of the purification methods herein reported.

Supplementary Figure S6; Table 1 indicates that treatment does not induce lattice parameter change or bulk structural rearrangement in the NMC, even in the presence of ionized metallic contaminants. It should be noted that the fraction of metallic contaminant added to these samples (1 wt%) is below the resolution of bulk XRD, and thus peaks attributable to the pure metal are not observed for the untreated condition.

**TABLE 1:** Rietveld refinement of samples shown in Figure 9.^a^

Condition	a (Å)^a^	c (Å)^a^	$\frac{I(003)}{I(104)}$	“R-factor”: $\frac{\{I(006)+I(012)\}}{I(101)}$
Pristine	2.86	14.23	1.05	.379
1% Al Spike, Untreated	2.86	14.23	1.20	.426
1% Al Spike, Treated	2.86	14.23	1.20	.401
1% Cu Spike, Untreated	2.86	14.23	1.20	.378
1% Cu Spike, Treated	2.86	14.23	1.16	.387

^a^ Lattice parameters are reported for the trigonal (R3̅m space group) phase, with estimated standard deviation (ESD) < 0.001 Å for all samples.

Finally, chemical analysis (ICP-MS) has been conducted to evaluate the efficacy of the treatment conditions in removing metallic contaminants in the practical context of an NMC matrix; results are presented in Table 2. The detection of residual Cu in the Cu-spiked sample after treatment initially appears to be inconsistent with titration and electrochemistry results; in particular, our baseline electrochemical studies with contaminated materials (Supplementary Figure S7) suggest that Cu induces electrochemical impacts at levels as low as 0.1 wt%. This disparity may in part be explained by the fact that the reported corrosion process acts relatively uniformly on all Cu particles, such that all particles are either fully corroded to Cu-DPTA or partially corroded and subsequently passivated with a stable, insulating surface species (e.g., Cu_2_O). The presence of pre-passivated and reduced-sized (i.e., <75 μm following partial corrosion) metallic Cu in NMC may minimize adverse impacts on electrochemical performance. Alternatively, it may be that solubilized bound Cu-DTPA was not fully removed during the post-treatment rinse and was present as a residue on the NMC surface, and the high stability of the chelated complex made the Cu electrochemically unreactive.

**TABLE 2:** ICP-MS analysis of contaminated NMC, following treatment; values are reported both as a raw mass percent (mass of metal per total sample mass) and as normalized by the total mass of transition metals (Co. + Mn + Li).

	Element mass%						Element mass fraction			
Sample condition	Li	Co.	Mn	Ni	Al	Cu	$\frac{Li}{Co+Mn+Ni}$	$\frac{Co}{Co+Mn+Ni}$	$\frac{Mn}{Co+Mn+Ni}$	$\frac{Ni}{Co+Mn+Ni}$
Pristine NMC-111	7.36	19.3	18.1	19.2	<0.004	.002	.130	.341	.320	.339
Treated 1% Al	7.16	18.8	17.3	18.5	<0.004	.002	.131	.344	.317	.339
Treated 1% Cu	7.17	18.9	17.4	18.6	<0.004	.214	.131	.344	.317	.339

The compositional data shown in Table 2 imply that the absolute mass percent of Li and transition metals is reduced for both batches of treated simulated BM, as compared to the pristine material. However, as indicated in the shaded columns of Table 2, the ratios of transition metals and the ratio of Li content vs. total transition metal content is consistent across treated vs. pristine material; this verifies that no transition metal or Li leaching has occurred. These ratios are identical between the two batches of treated material, which only varied in the presence of chelating agent (DTPA); this strengthens the conclusion that no bulk compositional change has occurred with treatment. The balance of mass accounting for the weight-percent differences between treated and pristine material is hypothesized to be residual treatment solvent species—primarily OH^−^ and K^+^—remaining on the NMC surface. The presence of residual K^+^ has previously been reported for delithiated cathode samples undergoing direct recycling treatment in a similar chemical matrix, i.e., treatment in KOH solution followed by a rinse in deionized water (Xu et al., 2021). Our preliminary EDS analysis on samples of shredded industrial battery scrap lends further support to this conclusion. While it is difficult to distinguish residual OH^−^ from the anticipated bulk lattice O in NMC, treated samples qualitatively show enrichment of the O signal relative to the signals of the lattice transition metals (Mn, Co., Ni) and relative to the O signal in the untreated material (Supplementary Figure S8). Further, the emergence of a K signal evenly dispersed across the treated sample strongly suggests the presence of residual treatment solvent, as there is no K present in the untreated sample. Thus, the ICP results are consistent with electrochemical results in highlighting the contribution of post-treatment conditions on the surface chemistry of the NMC.

As has been mentioned, development of a tailored post-treatment protocol to couple with the optimized bulk treatment parameters herein presented is crucial to the overall success of practical BM purification, and such studies are currently in progress. Continued concurrent elemental analysis will be used to verify that appropriate post-treatment conditions are sufficient to remove residual salts and recover the pristine elemental ratios. Despite the remaining challenges, the present BM purification process represents significant and crucial progress towards achieving high-quality directly recycled cathodes, particularly in cases where contamination is on the same order of size magnitude as the active material powder and cannot otherwise be removed.

## 4 Conclusion

Viable direct recycling of LIBs necessitates a high-purity output material, and the presence of metallic contaminants—including Al^0^ and Cu^0^—in the fine fraction of shredded black mass has proven detrimental to cathode performance. We have developed tailored methods to selectively ionize these contaminants using low-cost solvents and moderate temperatures, under conditions that maximize the rate of impurity removal while avoiding bulk structural and electrochemical damage to the target active material (NMC). In particular, we demonstrate that the input of kinetic energy through elevated temperature and sonication accelerates the corrosion of metallic Al and Cu under alkaline aqueous conditions, and 100% corrosion of each metal is achieved for 75 μm contaminant particles within 2.5 h at 60°C. We identify the importance of mass transport to facilitating rapid and complete Cu corrosion, and determine that the presence of saturated Cl^−^ in solution hinders rather than accelerates Cu corrosion; we primarily attribute this effect to the restricted mass transport induced by a highly viscous solution and competitive pathways for passivating surface film production on the corroding Cu. Evaluation of purification conditions on NMC implies no bulk structural damage, and electrochemical capacity is maintained in half-cell format. Full-cell testing suggests the presence of a limited quantity of residual surface species that are electrochemically consumed over initial cycling. We identify several plausible pathways to explain the evolution and observed reactivity of these species, whose adverse effect appears to be tied primarily to behavior at the graphite anode. By applying the reported BM purification process to samples of simulated BM—which contain a practically relevant 1 wt% Al or Cu contaminant and show catastrophic electrochemical performance—we are able to recover the electrochemical capacity of the pristine material. Efforts are underway to develop post-treatment conditions that optimize the surface chemistry and structure of treated material, so that this critical technique may be linked to subsequent processing in a direct recycling line.

## Data Availability

The raw data supporting the conclusion of this article will be made available by the authors, without undue reservation.
