# Supplementary material for: Optimized purification methods for metallic contaminant removal from directly recycled Li-ion battery cathodes
Source: Front Chem. 2023 Feb 8;11:1094198. doi: 10.3389/fchem.2023.1094198 (PMC9946041; doi:10.3389/fchem.2023.1094198)
Supplement: Supplementary file 1 [file DataSheet1.PDF]

Supplementary Material

1 Supplementary Figures and Tables

1.1 Supplementary Figures

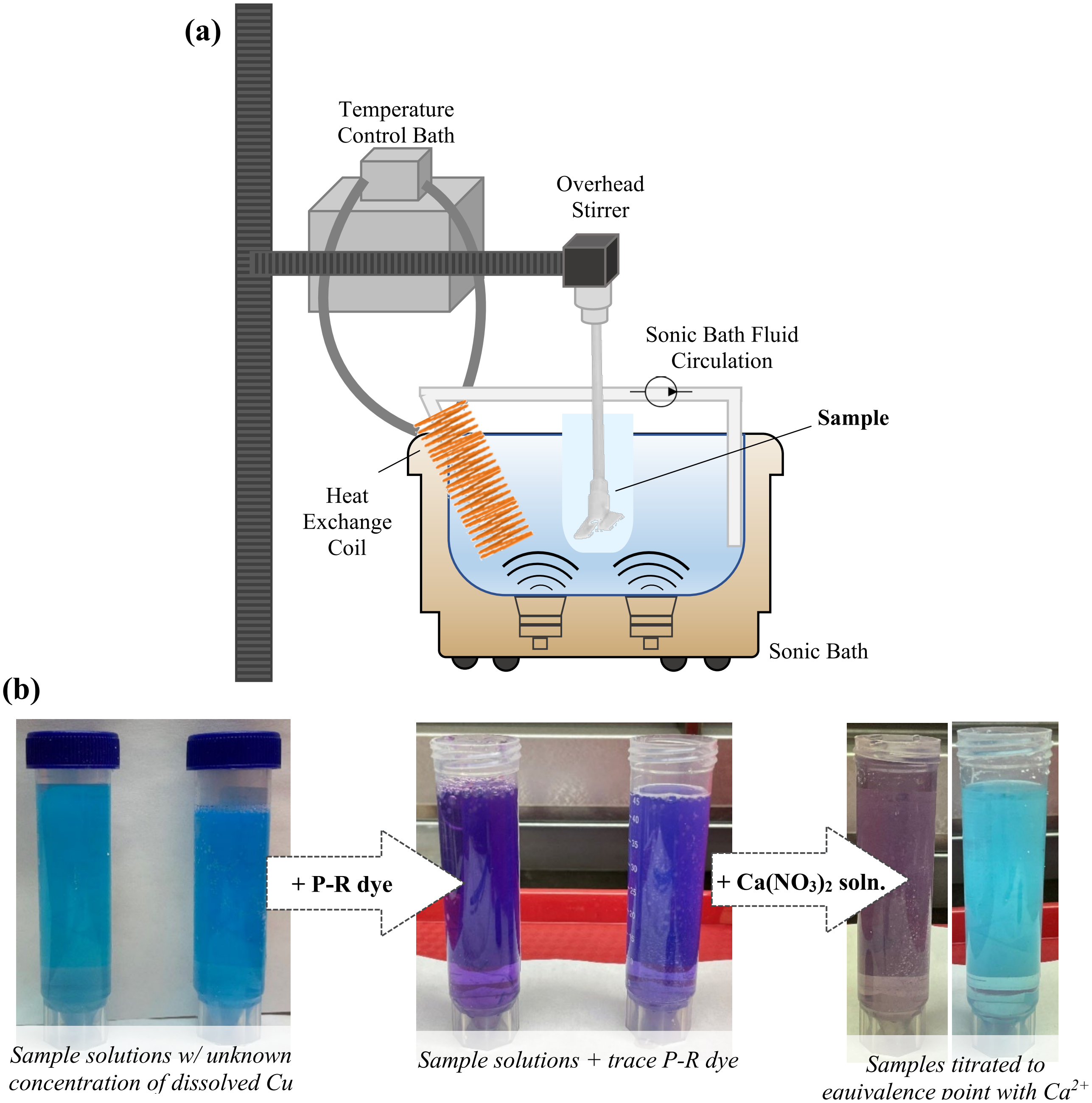

Supplementary Figure 1. (a) Schematic of sonic-stir bath developed for temperature-controlled, ambient-atmosphere testing with high-shear mixing and sonication; (b) Visual representation of colorimetric analysis approach for the quantification Cu.

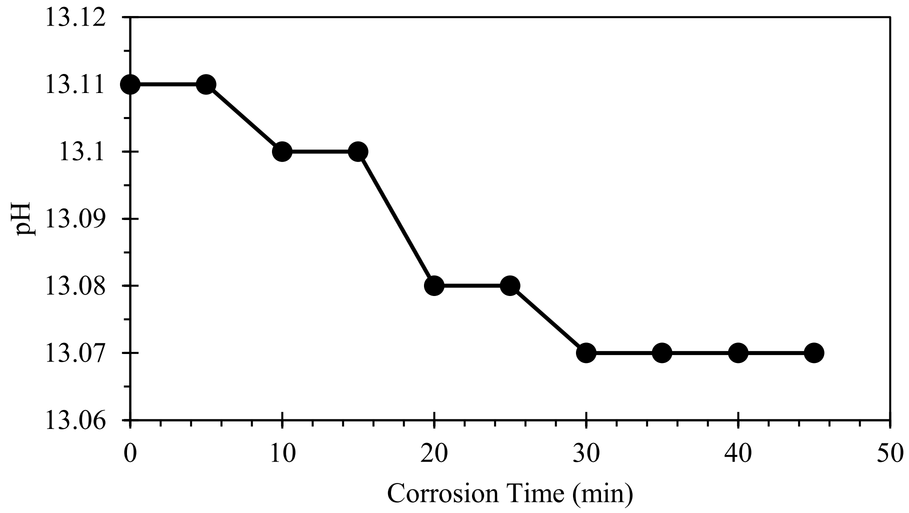

Supplementary Figure 2. Sample pH vs corrosion time data for $Al_{(s)}$ in KOH solution, with temperature maintained at constant calibration value (ambient room temperature). Data from this test is used to derive the theoretically calculated reduction in sample pH as $Al_{(s)} \rightarrow Al(OH)_4^-$, described below.

Measured Variables:
Initial mass H_2O: 40.0182 g
Initial mass $Al_{(s)}$: 0.0104 g
Initial pH (*as calibrated at test temperature*): 13.11

Calculated Variables:
Initial volume H_2O: 40.0182 mL ($\rho_{DI\ H_2O} = 1.000$ g/mL)
Initial moles $Al_{(s)}$: 0.000385 ($MW_{Al} = 26.982\frac{g}{mol}$)

Begin by calculating initial values for $[OH^-]$:

$$\begin{aligned}\textbf{Initial pOH} &= 14 - (\textbf{Initial pH})\\ &= 14 - 13.11\\ &= 0.89\end{aligned}$$

$$\begin{aligned}\textbf{Initial } [\mathbf{OH^-}] &= 10^{-(\textbf{Initial pOH})}\\ &= 10^{-0.89}\\ &= 0.1288\text{ M}\end{aligned}$$

$$\begin{aligned}\textbf{Initial moles } \mathbf{OH^-} &= (\textbf{Initial } [\mathbf{OH^-}]) \times (\textbf{Initial volume } \mathbf{H_2O})\\ &= \left(0.1288\frac{\text{mols}}{\text{L}}\right) \times 0.0400182\text{ L}\\ &= 0.005155\text{ mols OH}^-\end{aligned}$$

Complete (100%) corrosion of $Al_{(s)}$ to $Al(OH)_4^-$ consumes 1 stoichiometric equivalent of OH^- and 3 stoichiometric equivalents of H_2O (**Eq. (5)**, reproduced below):

$$Al_{(s)} + OH^- + 3H_2O \rightarrow Al(OH)_{4(aq)} + 3/2\ H_2$$

If 100% corrosion is achieved:

$$\textbf{Final moles OH}^- = (\textbf{Initial moles OH}^-) - (\textbf{Initial moles Al}_{(s)})$$
$$= (0.005155 - 0.000385)\text{ moles}$$
$$= 0.00477\text{ moles}$$

$$\textbf{Final volume H}_2\textbf{O} = (\textbf{Initial volume H}_2\textbf{O}) - \left(3 \times (\textbf{Initial moles Al}_{(s)}) \times MW_{H_2O} \times \rho_{DI\ H_2O}\right)$$
$$= 40.0182\text{ mL} - \left(3 \times (0.000385\text{ mols}) \times 18.013\frac{\text{g}}{\text{mol}} \times 1.000\frac{\text{g}}{\text{mL}}\right)$$
$$= 39.9974\text{ mL}$$

$$\textbf{Final } [OH^-] = {\textbf{\textit{Final moles OH}}^-}/{\textbf{\textit{Final volume H}}_2\textbf{\textit{O}}}$$
$$= {0.00477\text{ moles}}/{0.0399974\text{ L}}$$

$$= 0.1193\text{ M}$$

$$\textbf{Final pOH} = -\log[(\textbf{Final } [\textbf{OH}^-])$$
$$= -\log(0.1193\ M)$$
$$= 0.9235$$

$$\textbf{Final pH} = 14 - (\textbf{Final pOH})$$
$$= 14 - 0.9235$$
$$= 13.0765$$

Within instrumental accuracy limits (±0.01 mV), this matches the experimentally observed final pH value of 13.07 (as shown in **Fig. A.2**.), confirming that full corrosion is achieved at the point where the measured pH vs time curve reaches a stable plateau.

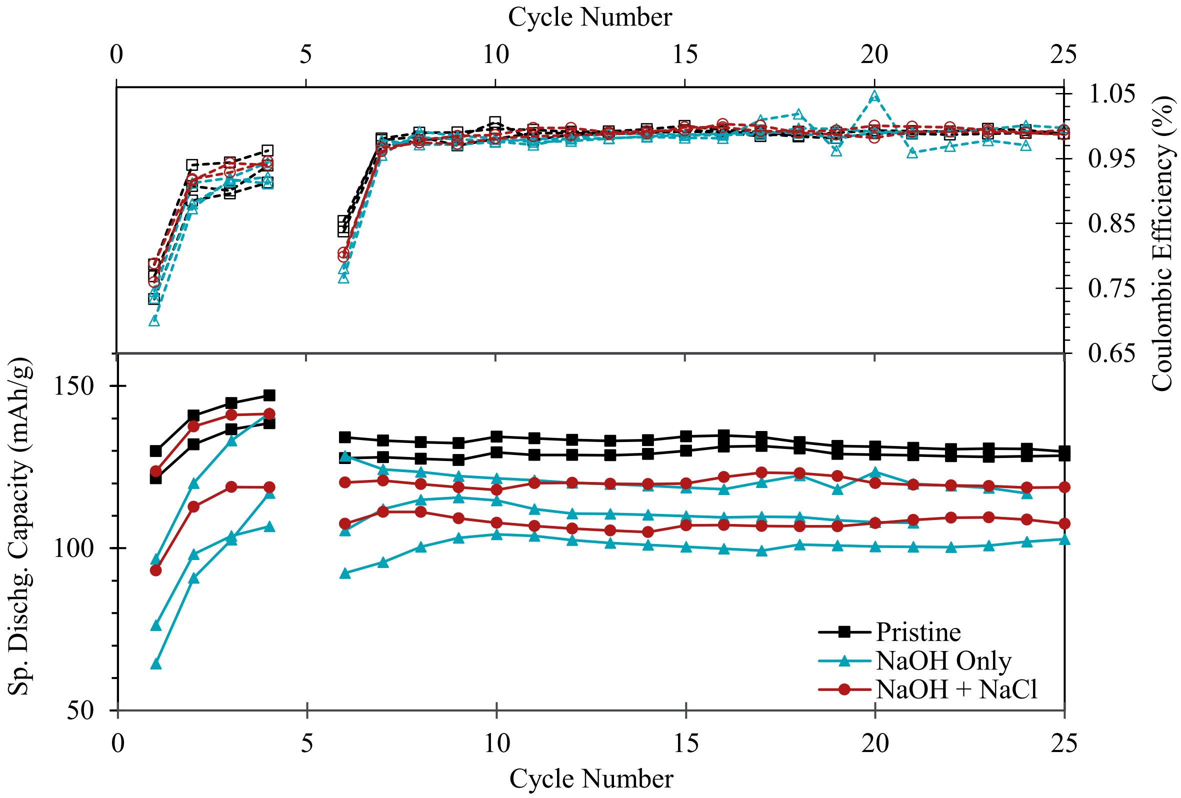

Supplementary Figure 3. Full-cell electrochemical performance of NMC-532 treated with Na^+ salts; significantly reduced capacity for treated material is attributed to Na^+/Li^+ exchange due to the similar ionic sizes between the two ions. This behavior prompted the reported shift to utilizing K^+-based salts.

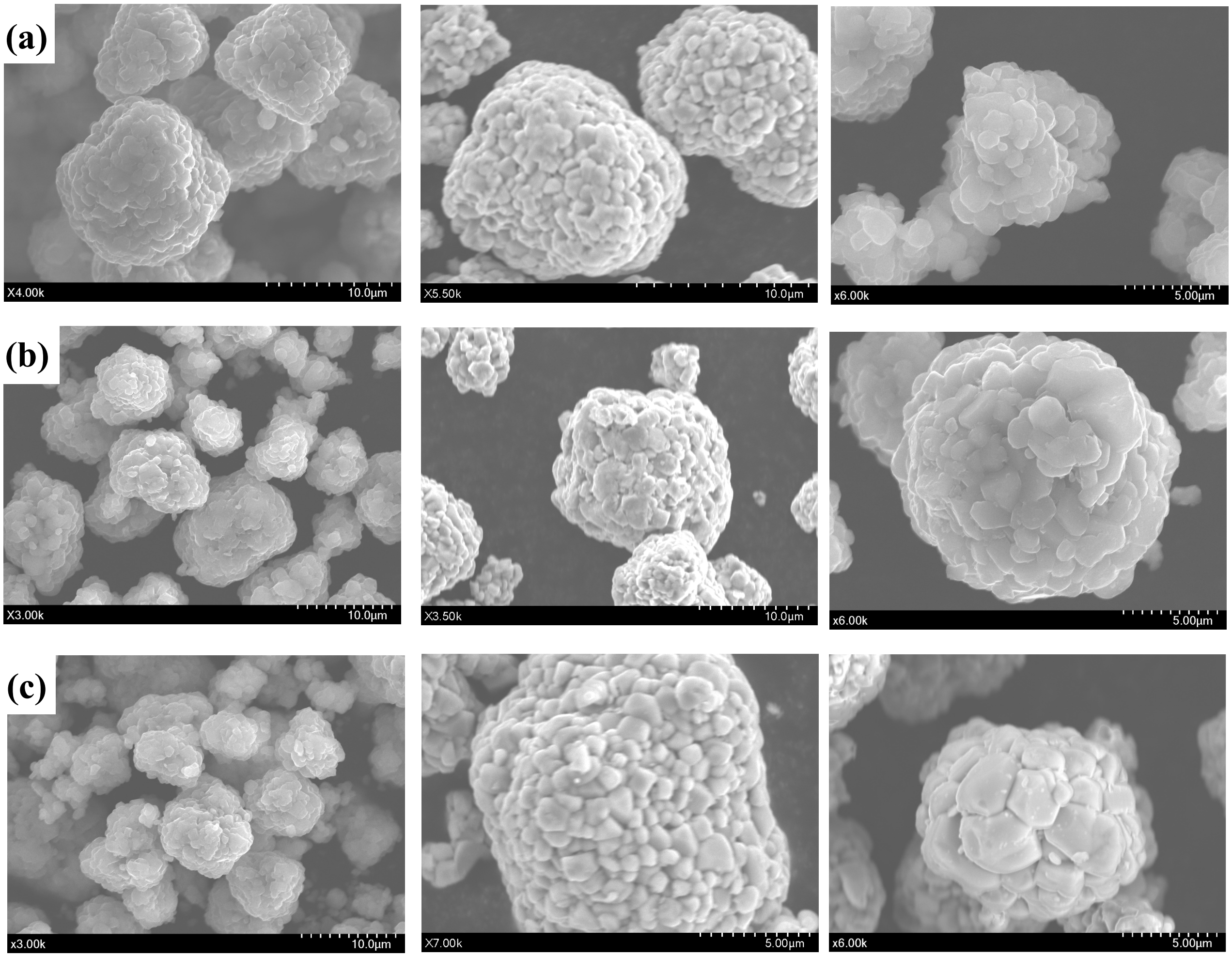

Supplementary Figure 4. Morphology of pristine and treated NMC-111; (a, top row): Pristine NMC-111; (b, middle row): KOH-treated NMC-111; (c, bottom row): KOH + KCl-treated NMC-111.

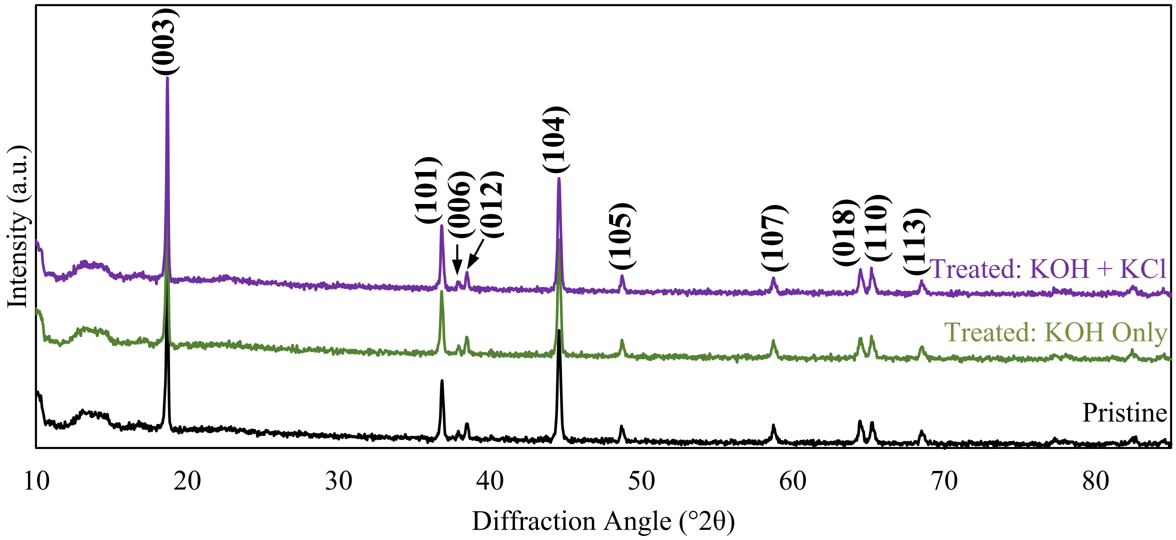

Supplementary Figure 5. Structural analysis of treated (KOH only (green) or KOH + KCl (purple)) NMC-111 versus pristine NMC-111 (black). All peak assignments are for trigonal ($R\bar{3}m$ space group) phase of NMC-111.

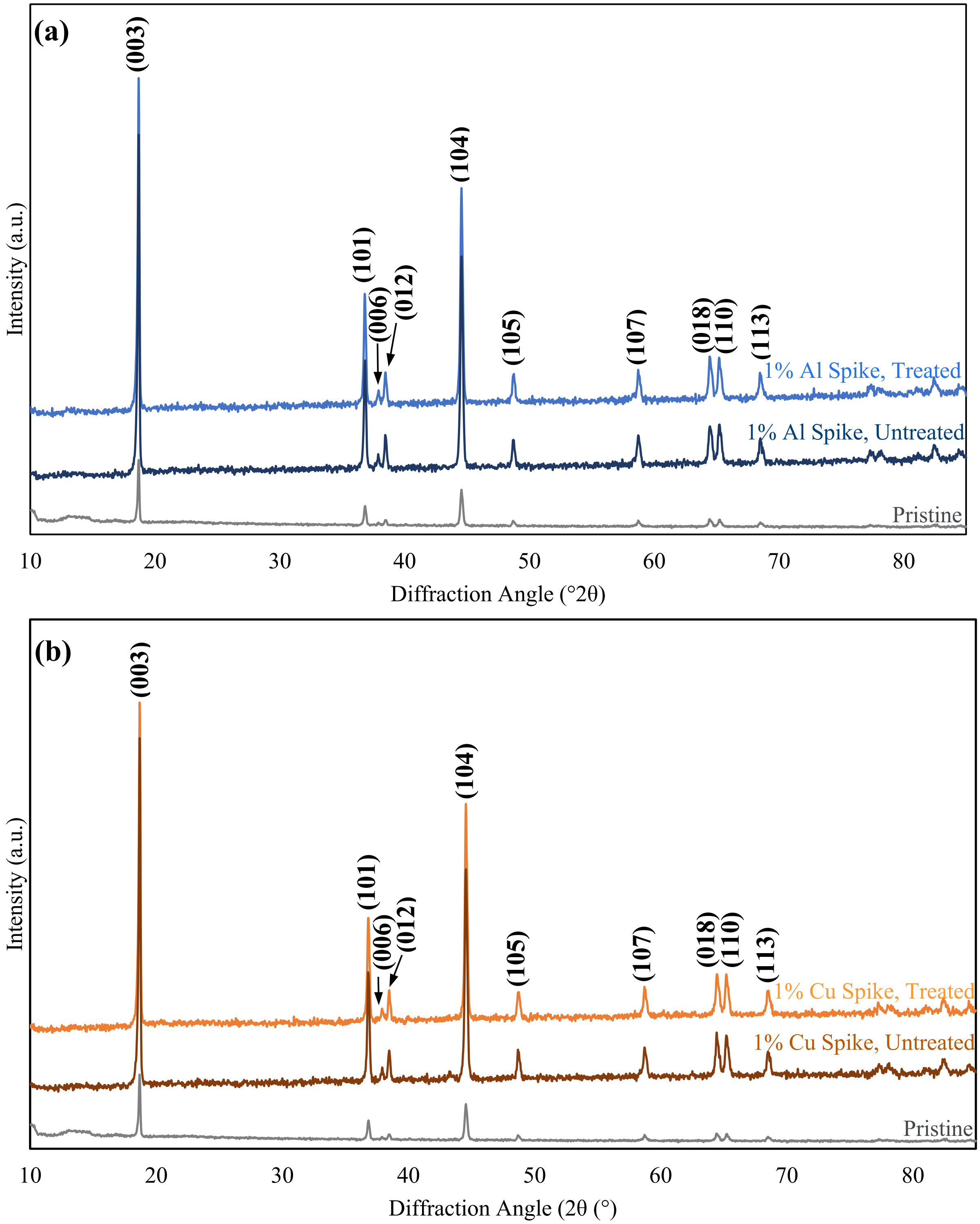

Supplementary Figure 6. Structural analysis of contaminated NMC-111, before and after treatment (vs pristine NMC-111): (a) Al-spiked NMC; (b) Cu-spiked NMC. All peak assignments are for trigonal ($R\bar{3}m$ space group) phase of NMC-111; as noted in text, the fraction of metallic contaminant added to these samples (1 wt%) is below the resolution of bulk XRD, and thus peaks attributable to the pure metal (Al or Cu) are not observed for the untreated condition.

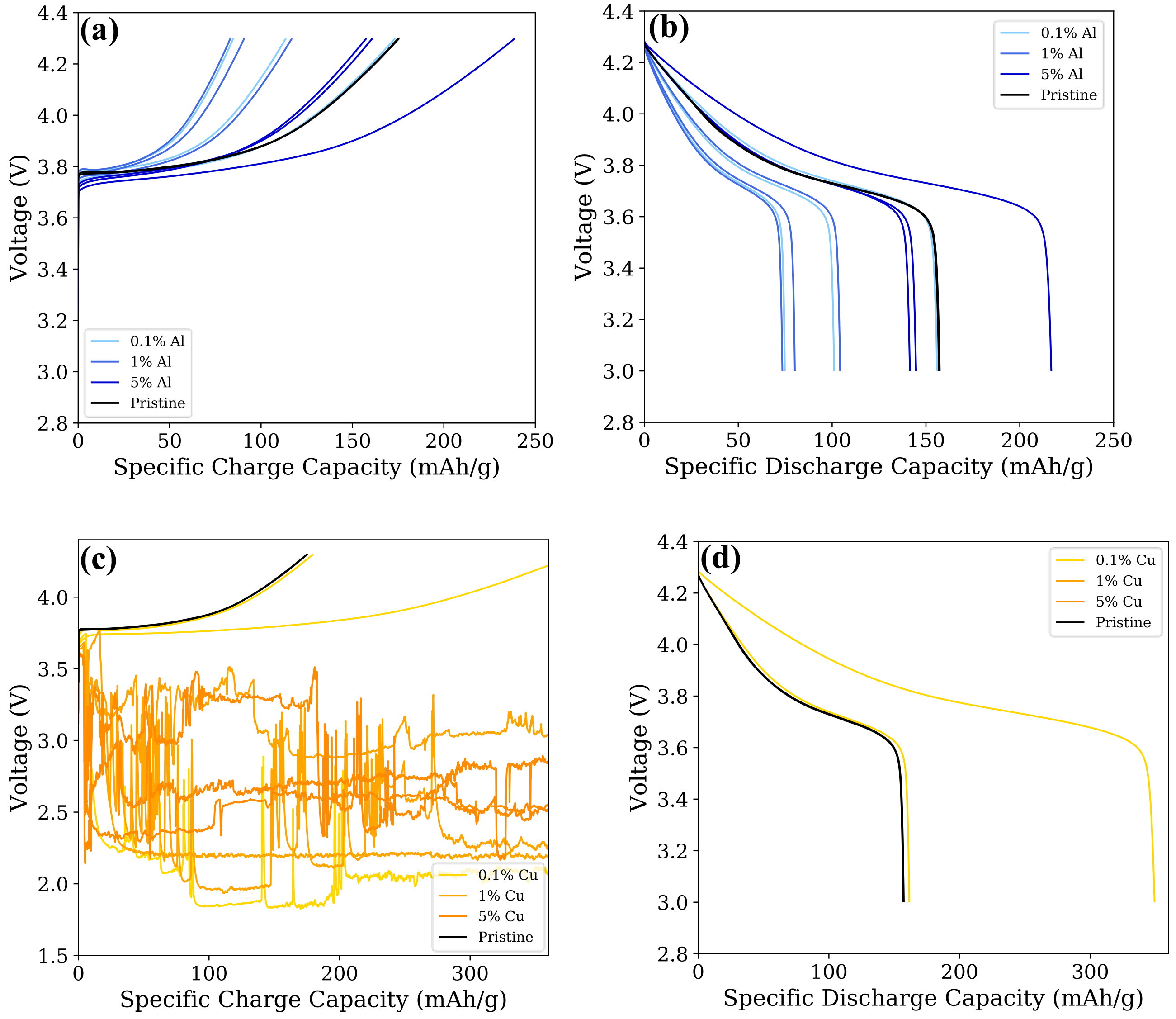

Supplementary Figure 7. First-cycle voltage profiles of NMC-111 spiked with various percentages of Al^0 (a-b) and Cu^0 (c-d) contaminant; all samples with >0.1% Cu displayed no discharge capacity. Note that first-cycle capacities for 0.1% Cu-spiked samples are artificially elevated due to continuous adverse reactivity; such capacity is irreversible upon continued cycling.

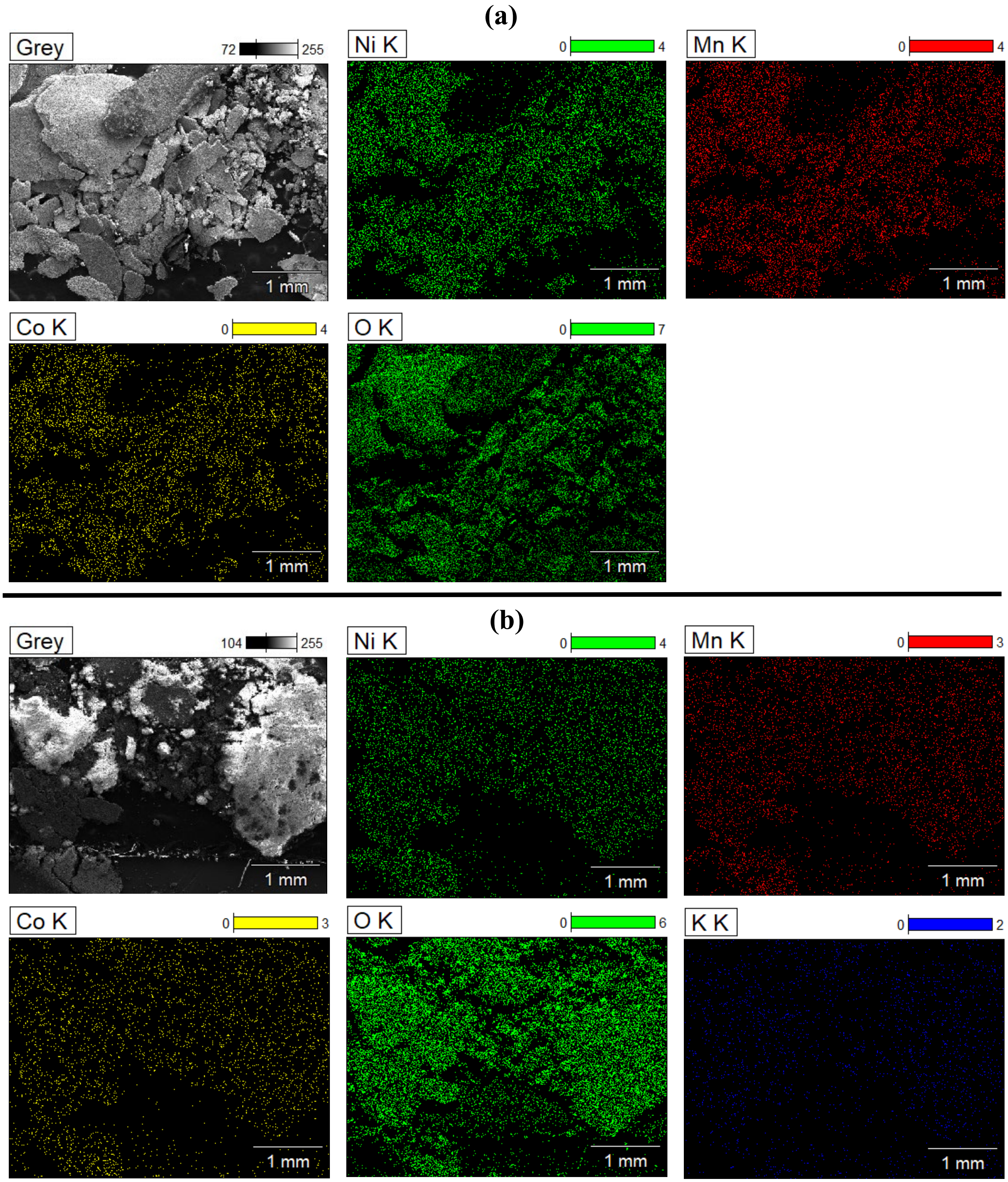

Supplementary Figure 8. EDS mapping of industrial black mass sample (a, top) before and (b, bottom) after black mass purification treatment; treated sample shows qualitative enrichment in O and K, suggesting incomplete removal of treatment solvent under unoptimized post-treatment rinse conditions. Untreated sample had no detectable K signal.

1.2 Supplementary Table

Table A1. Rietveld refinement of samples shown in Fig. A.5 showing calculated phase impurities; reference pattern sources are provided in the Methods section of the primary manuscript.

Condition	a (Å)	c (Å)	I(003)/I(104)	Relative Phase Percentage (% ± ESD)			
				α-NaFeO2 ($R\bar{3}m$)	Co_3O_4 ($Fd\bar{3}m$)	$LiNi_2O_4$ ($Fd\bar{3}m$)	NiO ($Fm\bar{3}m$)
Pristine	2.88	14.26	1.05	1.0 ± 0.7	5.0 ± 0.8	1.2 ± 0.7	0.8 ± 0.7
Treated: KOH Only	2.89	14.25	1.06	1.5 ± 0.9	4.0 ± 1.0	1.6 ± 0.7	2.0 ± 0.9
Treated: KOH + KCl	2.88	14.26	1.12	1.1 ± 0.5	4.6 ± 0.9	2.0 ± 0.8	1.6 ± 0.6
